# Contrasting controls on tree ring isotope variation for Amazon floodplain and terra firme trees

**DOI:** 10.1093/treephys/tpz009

**Published:** 2019-04-06

**Authors:** Bruno Barçante Ladvocat Cintra, Manuel Gloor, Arnoud Boom, Jochen Schöngart, Giuliano Maselli Locosselli, Roel Brienen

**Affiliations:** 1 School of Geography, University of Leeds, Leeds, Garstang North; 2 School of Geology, Geography and the Environment, Bennett Building, University Road, University of Leicester, Leicester, UK; 3 National Institute for Amazon Research, Av. André Araújo, 2.936, Petrópolis, CEP 69.067-375, Manaus, Amazonas Brazil; 4 Institute of Biosciences, University of São Paulo, Rua do Matão, 14, Butantã, São Paulo, CEP 05508-090, Brazil

**Keywords:** carbon isotopes, *Cedrela odorata*, dual isotope, *Macrolobium acaciifolium*, oxygen isotopes, tropical forests

## Abstract

Isotopes in tropical trees rings can improve our understanding of tree responses to climate. We assessed how climate and growing conditions affect tree-ring oxygen and carbon isotopes (δ^18^O_TR_ and δ^13^C_TR_) in four Amazon trees. We analysed within-ring isotope variation for two terra firme (non-flooded) and two floodplain trees growing at sites with varying seasonality. We find distinct intra-annual patterns of δ^18^O_TR_ and δ^13^C_TR_ driven mostly by seasonal variation in weather and source water δ^18^O. Seasonal variation in isotopes was lowest for the tree growing under the wettest conditions. Tree ring cellulose isotope models based on existing theory reproduced well observed within-ring variation with possible contributions of both stomatal and mesophyll conductance to variation in δ^13^C_TR_. Climate analysis reveal that terra firme δ^18^O_TR_ signals were related to basin-wide precipitation, indicating a source water δ^18^O influence, while floodplain trees recorded leaf enrichment effects related to local climate. Thus, intrinsically different processes (source water vs leaf enrichment) affect δ^18^O_TR_ in the two different species analysed. These differences are likely a result of both species-specific traits and of the contrasting growing conditions in the floodplains and terra firme environments. Simultaneous analysis of δ^13^C_TR_ and δ^18^O_TR_ supports this interpretation as it shows strongly similar intra-annual patterns for both isotopes in the floodplain trees arising from a common control by leaf stomatal conductance, while terra firme trees showed less covariation between the two isotopes. Our results are interesting from a plant physiological perspective and have implications for climate reconstructions as trees record intrinsically different processes.

## Introduction

Intra-annual, high-resolution oxygen and carbon isotopes are increasingly being used for a multitude of applications, including climate reconstructions (Barbour et al. 2002, Ohashi et al. 2009, Roden et al. 2009, Fichtler et al. 2010, Managave et al. 2011), age and growth rate determinations in ringless tropical trees (Poussart et al. 2004, Poussart and Schrag 2005, Pons and Helle 2011), and for studying seasonality in growth and use of carbohydrate reserves (Helle and Schleser 2004, Ohashi et al. 2009, Fichtler et al. 2010, Gulbranson and Ryberg 2013). A prerequisite for using tree-ring isotope records is an understanding of the underlying physiological processes affecting tree-ring isotope ratios.

Much progress in our understanding has been made over the past decades for temperate trees (McCarroll and Loader 2004). In comparison, isotope studies of tropical trees remain scarce, despite their great potential to improve our understanding of tree functioning and for climate reconstructions (van der Sleen et al. 2017) and the importance of these vast forests for the global carbon cycle (Phillips et al. 2009, Beer et al. 2010, Brienen et al. 2015, Pan et al. 2015). There is little information about what processes dominate variation of tree ring oxygen and carbon isotopes (δ^18^O_TR_ and δ^13^C_TR_) in tropical environments, and how this varies between different tropical tree species.

Oxygen isotope signals in tree rings are mostly the result of variation in source water δ^18^O and evaporative leaf enrichment (Dongmann et al. 1974, Roden and Ehleringer 1999, Farquhar et al. 2007, Cernusak et al. 2016). In tropical trees oxygen isotopes have been shown to reflect both processes (Miller et al. 2006, Brienen et al. 2011, Kahmen et al. 2011, Bowman et al. 2013, Schollaen et al. 2013), but which of these effects dominates and under which conditions remains poorly known. Specifically, the contribution of leaf water enrichment to the final δ^18^O_TR_ may vary between species and environments, due to variation in leaf transpiration arising from specific differences in leaf traits (e.g., varying pathlengths, Kahmen et al. 2008, Holloway-Phillips et al. 2016) and/or site humidity levels (Barbour and Farquhar 2000, Barbour et al. 2002, Kahmen et al. 2011).

Carbon isotope ratios in tree rings are affected by the ratio between photosynthetic assimilation rate and conductance to CO_2_. The conductance of CO_2_ from outside the leaf to the sites of photosynthesis consists of stomatal conductance, *g*_s_, and mesophyll conductance, *g*_m_, which both affect δ^13^C_TR_ (Farquhar et al. 1982, Seibt et al. 2008, Farquhar and Cernusak 2012). As *g*_s_ is often sensitive to water availability, δ^13^C_TR_ has been shown to reflect drought levels at relatively dry sites in the tropics (Gebrekirstos et al. 2009, Fichtler et al. 2010, Brienen et al. 2011). Mesophyll conductance, *g*_m_, is temperature dependent in many tree species and therefore variations in leaf temperature may also affect δ^13^C_TR_ (Seibt et al. 2008, Griffiths and Helliker 2013, von Caemmerer and Evans 2015). Other studies show that δ^13^C_TR_ signals also vary as a result of post-photosynthetic processes, specifically usage of carbon reserves (Helle and Schleser 2004, Eglin et al. 2010, Gulbranson and Ryberg 2013, Gessler et al. 2014).

**Figure 1.**
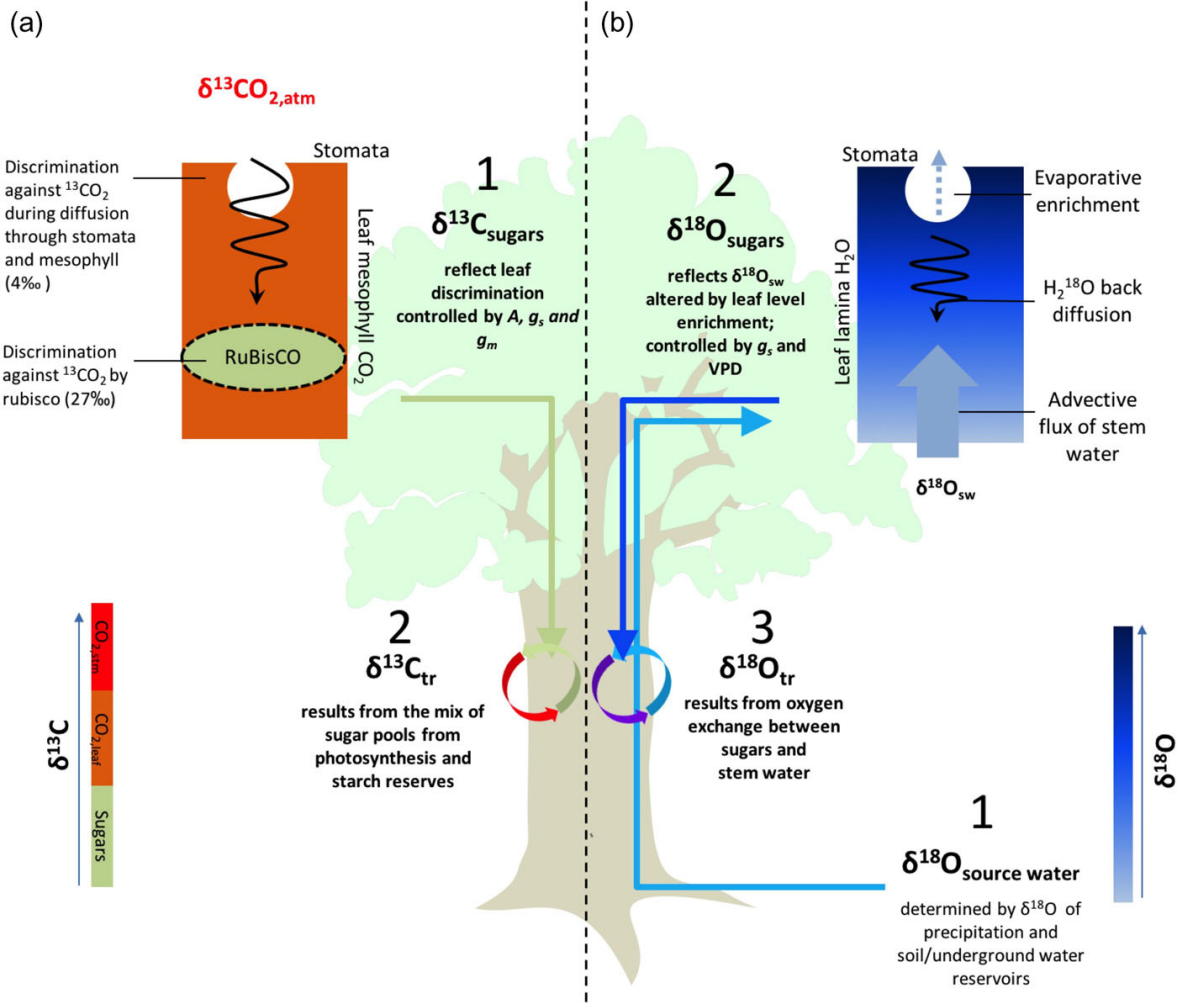
Illustration of the main processes driving isotope signals in tree rings. Left diagram (a) illustrates the two main processes affecting δ^13^C_TR_, which are (1) leaf level carbon isotope discrimination controlled by photosynthetic assimilation (*A*), stomatal and mesophyll conductance (*g*_s_ and *g*_m_, respectively), and (2) mixing of δ^13^C signals from leaf sugars and starch reserves into tree ring cellulose. Right diagram (b) illustrates the three processes affecting δ^18^O_TR_, which are (i) variation in source water δ^18^O, (ii) enrichment of the source water signal in the leaf due to evaporation and (iii) exchange of oxygen between stem water and sugars before incorporation into tree ring cellulose.

A useful approach to understand what processes are reflected in isotope signals is simultaneous analysis of variations of δ^18^O_TR_ and δ^13^C_TR_ in tree ring cellulose. This is because stomatal conductance response to low humidity may affect both leaf ^13^C discrimination and leaf water ^18^O enrichment, potentially leading to covariation of δ^13^C_TR_ and δ^18^O_TR_ (step 1 in Figure 1a and step 2 in Figure 1b). This approach has, for example, been used to assist in the interpretation of carbon isotope signals in leaves of temperate trees (Scheidegger et al. 2000), and allowed at least a partial separation of leaf level fractionation processes from the other fractionating effects on δ^18^O_TR_ and δ^13^C_TR_ (Barbour et al. 2002, Barnard et al. 2012, Roden and Farquhar 2012, Roden and Siegwolf 2012) (Figure 1).

**Figure 2.**
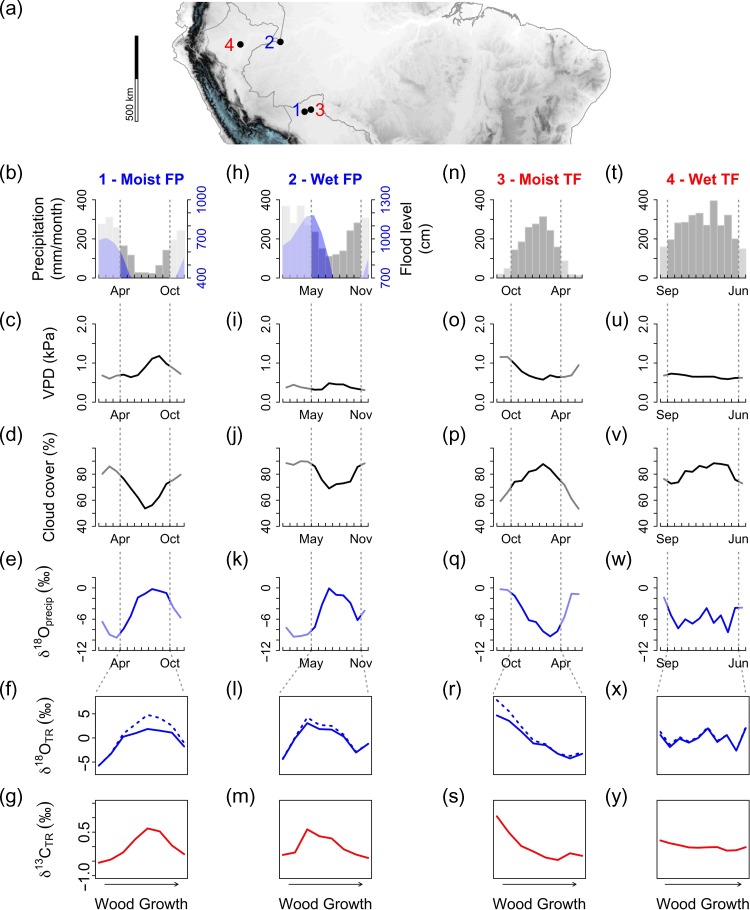
Map with the sampling sites (a) and annual cycles of climatic variables (b–e, h–k, n–q, t–w) and predicted intra-annual tree ring isotopes (f, g, l, m, r, s, x, y) for the two floodplain (FP) and the two terra firme (TF) sites. Annual cycle of monthly precipitation (grey bars) and river flood level (blue, showing levels above tree base) (top row), monthly VPD (second row), monthly cloud cover (third row), monthly δ^18^O in precipitation (fourth row), predicted δ^18^O in tree rings (solid line for effects of only source water δ^18^O and stippled line for added effects of leaf water enrichment) (fifth row) and predicted δ^13^C in tree rings, ignoring post-photosynthetic processes (lowermost row). The vertical dashed lines indicate the assumed growing seasons for each site—see details in section Data analysis. Climatic data shown are from CRU TS 4.00. Rainfall δ^18^O data are from the GNIP database. River level data are from the Brazilian Hidroweb-SNIRH database and from the ORE-HYBAM database for the Peruvian site.

Here we analyse which processes affect isotopic variation in tree rings of Amazonian trees, using a dual isotope approach with intra-ring resolution. This approach allows us to assess at a fine temporal scale how tree-ring isotopic compositions reflect the trees’ responses to varying weather conditions during the growing season. We chose two tree species growing under very different environmental conditions in the western and southwestern Amazon basin: the deciduous species *Cedrela odorata* L. (Meliaceae) from terra firme (non-flooded) forests that grow primarily during the wet season (Dünisch et al. 2003, Brienen et al. 2012, 2015, Costa et al. 2013, Baker et al. 2017), and the brevi-deciduous species *Macrolobium acaciifolium* (Benth.) Benth (Fabaceae) from floodplain ecosystems, which grows when river stage levels are low, i.e., during the dry season (Schöngart et al. 2002, 2005, Assahira et al. 2017). The original motivation for looking at these contrasting environments was that terra firme trees would record wet season and floodplain dry season climate variation. We chose these species because both are spatially widespread (ter Steege et al. 2013), grow in contrasting conditions and produce distinct annual rings (see Figure S1 available as Supplementary Data at *Tree Physiology* Online). For both species, we investigate two trees from two sites at a high intra-ring resolution (four trees in total). The sites were selected to differ in precipitation amount and seasonality (Figure 2).

Our objectives are: (i) to compare observed and expected intra-annual patterns of carbon and oxygen isotope within their tree rings based on existing mechanistic understanding; (ii) to assess the role of local climate and hydrological conditions and species differences on the intra-annual cycles of δ^18^O_TR_ and δ^13^C_TR_; and (iii) to assess to what degree the dual-isotope approach may indicate what the main climatic and physiological drivers of variation in both isotopes at the intra-annual level are.

### Isotopes in tree rings: theory and modelling

#### Carbon isotopes

Atmospheric CO_2_ is the source of carbon for terrestrial trees. During CO_2_ uptake by a leaf, four fractionating processes are of importance: CO_2_ diffusion through the stomata and through the leaf mesophyll (i.e., cell membranes and cytoplasm), photorespiration and isotopic fractionation during the carboxylation reaction due to higher chemical affinity of the enzyme RuBisCO for ^12^CO_2_ compared with ^13^CO_2_ (step 1 in Figure 1a). These processes result in lower average plant organic ^13^C compared with the atmosphere, or a positive atmosphere–plant isotope discrimination (Δ)

(1) $${∆}^{13}C\equiv \frac{R_{\mathrm{atm}}-R_{\mathrm{plant}}}{R_{\mathrm{plant}}}\cdot 10^{3}=\frac{\delta^{13}C_{\mathrm{atm}}-\delta^{13}C_{\mathrm{plant}}}{1+\frac{\delta^{13}C_{\mathrm{plant}}}{1000}}.$$

Here $R\equiv \frac{N_{\mathrm{rare}}}{N_{\mathrm{abundant}}}$ with *N*_rare_ the number of molecules of the rare isotope compound and *N*_abundant_ the number of molecules of the abundant isotope compound in a sample, and with $\delta \left(\mathrm{permille},‰\right)\equiv \left(\frac{R_{\mathrm{sample}}}{R_{\mathrm{std}}}-1\right)\cdot 10^{3}$, where *R*_std_ is the isotope ratio of an internationally recognized standard. Farquhar et al. (1982) formulated a model to predict this discrimination that considers fractionation during diffusion through stomata and through the mesophyll, during carboxylation and fractionation due to photorespiration:

(2) $${∆}^{13}C=a\left(\frac{c_{a}{-c}_{i}}{c_{a}}\right)-a_{m}\left(\frac{c_{i}{-c}_{c}}{c_{a}}\right)+b\left(\frac{c_{c}}{c_{a}}\right)-f\Gamma^{⁎}/c_{\mathrm{a,}}$$

where *c*_i_ is CO_2_ partial pressure inside leaf intercellular space, *c*_a_ is CO_2_ partial pressure in air, *c*_c_ is the CO_2_ partial pressure in the chloroplast, a (=4.4‰) is the fractionation caused by slower diffusion of ^13^CO_2_ compared with ^12^CO_2_ through stomata, *a_m_* (=1.8‰) is the fractionation during CO_2_ diffusion through the mesophyll, *b* (=30‰) is fractionation during carboxylation caused by discrimination of RuBisCO against ^13^CO_2_ inside the leaf, *f* (=12‰) is the discrimination due to photorespiration and Γ* is the CO_2_ compensation point in the absence of day respiration (see also Seibt et al. 2008).

The model predicts that if *c*_c_ is close to *c*_a_, then discrimination is primarily due to non-equilibrium fractionation associated with carboxylation (≈i). If, on the other hand, CO_2_ in the leaf is being drawn down by assimilation and *c*_c_ drops, then the carboxylation reaction causes an increase of the ^13^CO_2_ to ^12^CO_2_ ratio inside the leaf (a Rayleigh distillation, step 1 in Figure 1a). The consequent enrichment of CO_2_ inside the leaf offsets the effects of fractionation by carboxylation and lowers the net discrimination slightly towards the value for fractionation by diffusion (≈a). For plants, the magnitude of fractionation thus depends on the CO_2_ partial pressure difference *c*_a_*– c*_c_ between the outside and inside of the leaf. This difference is controlled by the ratio between carbon assimilation rate (*A*) and CO_2_ flux in the leaf via $A=g_{\mathrm{sC}}\left(\frac{c_{a}-c_{i}}{P}\right)=g_{m}\left(\frac{c_{i}-c_{c}}{P}\right)$, which expresses that at steady state, assimilation rate *A* is equal to diffusive CO_2_ flow though stomata and into the chloroplast. Here *P* is atmospheric air pressure, *g*_sC_ is the stomatal conductance to CO_2_ and *g*_m_ is the mesophyll conductance to CO_2_. Equation (2) may then be expressed as

(3) $${∆}^{13}C=a+\left(b-a\right)\frac{c_{i}}{c_{a}}-\left(b-a_{m}\right)\left(\frac{A}{c_{a}g_{m}}\right)-f\Gamma^{⁎}/c_{a}.$$

Isotope ratios of sugars produced in the leaf may undergo alterations before being incorporated in wood tissue by processes such as respiration, re-fixation of respired CO_2_, and production and remobilization of carbon reserves, primarily starch (e.g., Cernusak et al. 2009, Treydte et al. 2014). In particular, for some deciduous trees wood formation before leaf flush requires the mobilization of non-structural carbohydrate reserves (NSC, step 2 in Figure 1a). Non-structural carbohydrate reserves that have accumulated by the end of the previous growing seasons are usually enriched in ^13^C in comparison with new photosynthesis assimilates (Brugnoli et al. 1988, Damesin and Lelarge 2003). Thus the use of stored NSC during the growing season may lead to higher δ^13^C in initial tree ring sections (Helle and Schleser 2004, Skomarkova et al. 2006, Ohashi et al. 2009, Gulbranson and Ryberg 2013, Gessler and Treydte 2016), and possibly in other ring sections as well (Eglin et al. 2010). These post-photosynthetic processes may partially decouple the δ^13^C_TR_ signal from current years’ leaf fractionation processes, potentially dampening the climatic signal in δ^13^C_TR_.

In summary, two processes contribute to intra- and inter-annual variation in δ^13^C_TR_: (i) ^13^C discrimination during leaf carbon uptake and photosynthesis; and (ii) carbon remobilization from non-structural carbon reserves. δ^13^C_TR_ derived from reserves is enriched with ^13^C and for deciduous species tends to be used primarily during the initial phase of tree ring formation. ^13^C discrimination at the leaf level is controlled by the ratio of CO_2_ inside the leaf to CO_2_ in air. If this ratio is low—either because of low stomatal/mesophyll conductance to CO_2_ associated with high vapour pressure deficit (VPD) or low temperatures, or due to high assimilation rates—discrimination will be small and vice versa.

#### Oxygen isotopes

More processes contribute to δ^18^O variation in tree ring cellulose (δ^18^O_TR_) compared with δ^13^C_TR_. First, δ^18^O_TR_ is related to the isotopic composition of source water (δ^18^O_sw_), which may originate from rainfall and/or from underground water (step 1 in Figure 1b). δ^18^O_sw_ may change in the soil by fractionation during evaporation. Water is taken up from the soil by roots without fractionation (Ehleringer and Dawson 1992). Xylem water entering the leaf has thus the same δ^18^O as soil water. In the leaf, water will get enriched in H_2_^18^O compared with stem water due to preferential evaporation of light water, H_2_^16^O (Craig and Gordon 1965, Dongmann et al. 1974). Average leaf water δ^18^O (δ^18^O_lw_) depends on the extent of ^18^O enrichment of water at the sites of evaporation within the leaves (δ^18^O_es_), and on how much H_2_^18^O diffuses from the sites of evaporation through the leaf lamina, which depends on transpiration (Farquhar and Lloyd 1993, Farquhar et al. 2007, Cernusak and Kahmen 2013). Transpiration is driven by leaf to air vapour pressure difference (VPD) modulated by stomatal conductance (which itself may depend on VPD) (step 2 in Figure 1b). Sugars produced in the leaf carry with them the ^18^O-enriched leaf water signal (δ^18^O_lw_) until they are broken down during cellulose synthesis, when they exchange oxygen isotopes with water in the stem (step 3 in Figure 1b).

The roles of the above-mentioned processes have been incorporated into models. The earliest model for δ^18^O at the leaf sites of evaporation is from Dongmann et al. (1974) based on a model of Craig and Gordon (1965) for fractionation during the process of evaporation from a water surface. The Dongmann model considers a water flow from roots to the stomata to the atmosphere, equilibrium fractionation ε^+^ during evaporation from tissue in the stomata and kinetic fractionation ε_k_ during diffusion of molecules from the leaf to the atmosphere through stomata. The resulting model for the isotopic signature at the site of evaporation δ^18^O_es_ is (same as Eq. (1) of Sternberg 2009, but algebraically rearranged):

(4) $$\delta^{18}O_{\mathrm{es}}=\left(\delta^{18}O_{\mathrm{sw}}+\varepsilon_{k}\right)\left(\frac{e_{i}-e_{a}}{e_{i}}\right)+\varepsilon^{+}+\delta^{18}O_{a}\left(\frac{e_{a}}{e_{i}}\right).$$

*e*_i_ and *e*_a_ are the intracellular and ambient vapour pressure, respectively; $\varepsilon_{k}=\frac{32g_{s}^{-1}+21g_{b}^{-1}}{g_{s}^{-1}+g_{b}^{-1}}\sim 26.5‰$ is the kinetic isotopic fractionation of diffusion through stomata and boundary layer, *g*_b_ is the leaf boundary layer conductance (Farquhar et al. 1989); $\varepsilon^{+}=2.644-3.206\left(\frac{10^{3}}{K}\right)+1.534\left(\frac{10^{6}}{K^{2}}\right)=9.57‰$ (at 20 °C) is the temperature-dependent isotopic equilibrium fractionation between vapour and water at the evaporation site inside the stomata, where *T* is the leaf temperature in Kelvin. At higher temperatures ε^+^ tends to decrease, but this effect is relatively small (Bottinga and Craig 1969).

**Figure 3.**
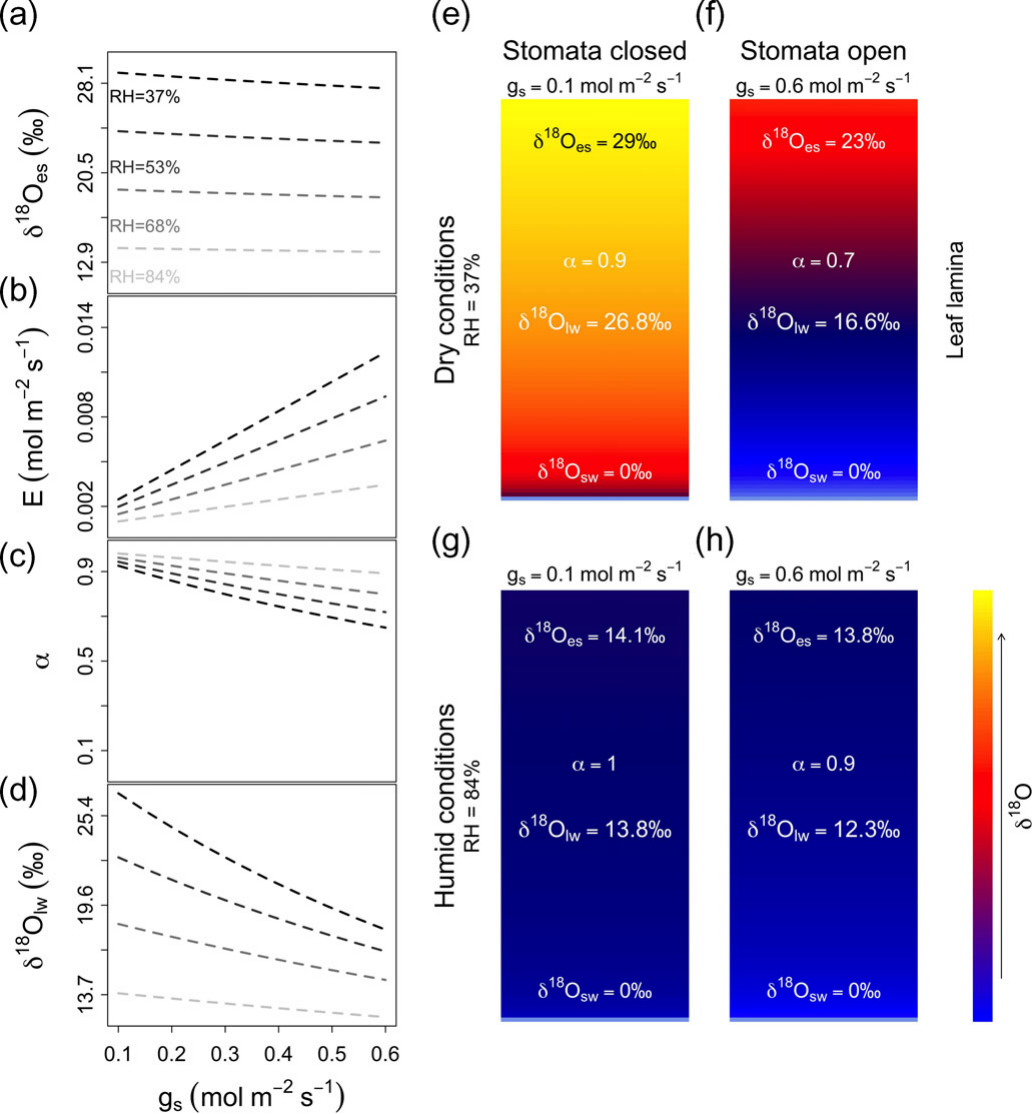
Predicted effects of stomatal conductance (*g_s_*) on leaf water δ^18^O at four different levels of relative humidity (RH). Modelled relationship between *g_s_* and δ^18^O of water at the sites of evaporation (δ^18^O_es_) (a), *g_s_* and leaf transpiration (*E*) (b), *g_s_* and ‘mixture’ of source water with water from the sites of evaporation, $\mathrm{alpha}=\left(\frac{\left(1-e^{-℘}\right)}{℘}\right)$, (c), and *g*_s_ and mean leaf water δ^18^O (δ^18^O_lw_) (d). Panels (e)–(h) illustrate predicted δ^18^O gradients between incoming leaf water (δ^18^O_sw_) and δ^18^O at sites of evaporation (δ^18^O_es_) for low relative humidity (top panels) and high relative humidity (lower panels), and for low (left panels) and high stomatal conductance (right panels).

To interpret the model it is helpful to express leaf transpiration through stomata as $E=\mathrm{gs}*\frac{e_{i}-e_{a}}{P}=\mathrm{gs}*\frac{\mathrm{VPD}}{P}$ where $\mathrm{VPD}\equiv e_{i}-e_{a}$ is leaf to air vapour pressure difference (or ‘deficit’) and *P* is the atmospheric pressure. Thus, if VPD ~ 0, there is no leaf to air water flow and thus no flow from the stem into the leaves. In this case, δ^18^O_es_ is just the sum of atmospheric δ^18^O_a_ (*e*_i_/*e*_a_ = 1) and the equilibrium fractionation ε^+^ of evaporation inside the stomata. If in contrast there is a flow of water from the leaf via stomata to the atmosphere (i.e., when VPD > 0) and thus also a flow of stem (source) water to the stomata, then there is also a contribution to δ^18^O_es_ from source water δ^18^O_sw_ and from kinetic fraction ε_k_ during diffusion of water molecules through the stomatal opening (see Figure 3a).

The Dongmann et al. (1974) model described above tends to overestimate leaf water δ^18^O. Farquhar and Lloyd (1993) suggest that this is because average leaf water δ^18^O is a mixture of δ^18^O at the evaporative site (δ^18^O_es_) and in the source water (δ^18^O_sw_). The relative contribution of δ^18^O_es_ and δ^18^O_sw_ to average leaf water δ^18^O (δ^18^O_lw_) depends on the degree of back-diffusion of isotopes from the evaporation site along the water-stream from veins to stomata. The lower the water flow, the more important is the effect of back-diffusion and vice versa. The importance of back-diffusion can be measured by the Péclet number

(5) $$℘\equiv \frac{\mathrm{Advection}}{\mathrm{Diffusion}}=\frac{u}{\frac{D}{L}},$$

the ratio of advective water transport (velocity *u*, which can be expressed using transpiration as $u=\frac{E}{C}$ where *c* is concentration of water, e.g., in mol m^−3^) by the water stream from soil to air via stomata and the counteracting diffusive transport (*D* molecular diffusivity of water and *L* the length of the path from stomata into leaf veins) (see also Cernusak and Kahmen 2013). Farquhar and Lloyd (1993) formulated a model of this effect that predicts δ^18^O in the leaf within a distance *L* along veins from the evaporative site:

(6) $$\delta^{18}O_{\mathrm{lw}}=\delta^{18}O_{\mathrm{sw}}+\left(\delta^{18}O_{\mathrm{es}}-\delta^{18}O_{\mathrm{sw}}\right)⁎\frac{\left(1-e^{-℘}\right)}{℘}$$

The term $\alpha =\frac{\left(1-e^{-℘}\right)}{℘}$ determines the contribution of δ^18^O_es_ to δ^18^O_lw_, resulting from back diffusion. According to this model, if transport via advection of stem water to the evaporation site is much faster than the counteracting diffusive transport—i.e., when ℘ is large—δ^18^O of leaf water is close to δ^18^O of source (stem) water. If, in contrast, transport by advection of stem water is less than by back-diffusion, then δ^18^O of leaf water will be close to δ^18^O_es_$\left(\mathrm{since}\frac{\left(1-e^{-℘}\right)}{℘}\to 1\mathrm{for}℘\to 0\right)$ (Figure 3b and d). Thus, because the advective flux is given by transpiration, *E*, the contribution of δ^18^O_es_ to δ^18^O_lw_ is controlled by both stomatal conductance to water, *g*_sW_ and VPD (Figure 3b–d). Since transpiration is linearly proportional to VPD, small changes in *g*_sW_ have a large effect on net transpiration (and thus on δ^18^O_lw_) when VPD is large (see Figure 3).

Finally, tree ring cellulose δ^18^O (δ^18^O_TR_) depends on post-photosynthetic fractionation, which occurs when sugars exchange oxygen with stem water during cellulose synthesis (step 3 in Figure 1b). According to Sternberg (2009) this fractionation can be parameterized as:

(7) $$\delta^{18}O_{\mathrm{tr}}=\phi \left(\delta^{18}O_{\mathrm{sw}}+\Delta \right)+\left(\left(1-\phi \right)*\left(\delta^{18}O_{\mathrm{sub}}\right)\right)$$

Here ϕ (~0.4) is the proportion of oxygen from sugars that exchanged with stem water during this process, Δ is the average fractionation of the oxygen that exchanged with stem water and δ^18^O_sub_ is the δ^18^O of sugars that did not exchange with water during cellulose synthesis. Therefore, ϕ tends to reinforce the source water δ^18^O signal in tree ring cellulose, without completely erasing the leaf water enrichment signal. Experiments have shown that ~40% of the sugars exchange oxygen with stem water before incorporation into cellulose, with variations between different tree species (DeNiro and Cooper 1989, Luo and Sternberg 1992, Cernusak et al. 2005, Sternberg et al. 2006).

In summary, three processes control δ^18^O_TR_: (i) source water δ^18^O; (ii) enrichment of source water in the leaf during evaporation, which depends on VPD and leaf transpiration rates (see Figure 3); and (iii) the degree of exchange of oxygen in exported sugars with stem water during cellulose synthesis. The degree of leaf enrichment increases linearly with increasing VPD, but also depends on transpiration rate (due to back-diffusion), and is thus related to *g*_sW_ and VPD. The sensitivity of leaf enrichment to *g*_sW_ is predicted to be highest under high VPD or low relative humidity (see Figure 3).

We thus expect that intra-annual variation of δ^18^O_TR_ will be primarily influenced by the seasonal cycle of source water or precipitation δ^18^O, which varies quite strongly over trees’ growing season (Figure 2e, k, q and w). Leaf level enrichment processes will add to this ‘background’ variation by causing enrichment, which is expected to be greater under higher VPD and expected to be more strongly modulated by *g*_sW_ under drier conditions (see Figure 3).

## Materials and methods

### δ^18^O_TR_ and δ^13^C_TR_ predictions

To make our expectations more quantitative we have used the tree ring-isotope models described in the section Isotopes in tree rings: theory and modelling to predict the sensitivity of intra-annual variation in both isotopes to weather conditions during the trees’ growing seasons. Climatic variables that influence these predictions were VPD, temperature and source water δ^18^O. Our predictions also depend on estimated responses to *g*_s_ and *g*_m_ to the climatic variables. For both isotopes, stomatal conductance to water and CO_2_ were calculated as a function of VPD via $g_{\mathrm{sW}}=1.6g_{\mathrm{sC}}=g_{\mathrm{sMax}}\left(\frac{1}{1+\left(\frac{\mathrm{VPD}}{{\mathrm{VPD}}_{\mathrm{mean}}}\right)}\right)$. Here *g*_sMax_ (0.5 mol m^2^ s^−1^) is an assumed value for maximum *g*_sW_ and VPD_mean_ is the long-term mean of VPD during the growing season of each tree. For δ^13^C_TR_, *g*_m_ was estimated as a linear function of temperature via $g_{m}=g_{m25}\left(0.44+0.058T\right)$ (Evans and von Caemmerer 2013), where *g*_m25_ is the *g*_m_ at 25 °C and *T* is temperature in Celsius. As *g*_m_ is highly variable between species (von Caemmerer and Evans 2015) and we have no information about *g*_m_ for either of the species in this study, we considered an assumed of *g*_m25_ = 0.19 mol m^2^ s^−1^ for both species. For δ^13^C_TR_ predictions, we ignored carbon remobilization, as we lacked sufficient insight to quantify these processes. We also did not consider any seasonal variations in the growth rates of the trees, as we lack information on growth rhythm during the growing season for the two tree species at the study sites. Further details of the models and parameters used for predictions of δ^18^O_TR_ and δ^13^C_TR_ can be found in Table S2 available as Supplementary Data at *Tree Physiology* Online.

### Sites and species selection

#### Terra firme sites and species

Two terra firme sites (i.e., non-flooded) differing in total annual rainfall were selected for this study (Figure 2a); a wet site in the Peruvian Amazon with total annual precipitation of 2500 mm (−4° 54′ 00″ N, −73° 47′ 59.62″E), and a moist site in the Bolivian Amazon with 1700 mm annual precipitation (−10° 59′ 60″N, −65° 00′ 00″E). Precipitation in the moist site is highly seasonal, dropping below 100 mm per month for up to 5 months per year, while mean monthly precipitation in the wet terra firme site rarely drops below 100 mm.

The species we choose for terra firme forests, *C. odorata*, grows during the wet season and stops growing at the onset of the dry season, when it sheds its leaves (Dünisch et al. 2003, Costa et al. 2013). New leaf flush occurs several weeks later at the end of the dry season (Dünisch et al. 2003, Brienen and Zuidema 2005). Previous studies on *C. odorata* from the south west of the Amazon basin have shown that δ^18^O_TR_ reflects rainout processes upwind of the growth site, and thus are a good proxy for basin-wide rainfall in the Amazon (Brienen et al. 2012, Baker et al. 2016).

#### Seasonal floodplain sites and species

Várzea forests are one of the most representative seasonal floodplain forests, supporting annual flooding of up to 7 m (Wittmann et al. 2012). Two várzea floodplain sites were selected, a ‘wet floodplain site’ in the Colombian Amazon receiving 2600 mm of annual precipitation (−4° 30′ 00″N, −70° 00′ 00″E), and a ‘moist site’ in the Bolivian Amazon receiving 1700 mm of annual rainfall (−11° 33′ 30″N, −67° 18′ 54″E).

*Macrolobium acaciifolium*, the species chosen for this environment, renews its canopy during the flooded period, which lasts for ~6 months, after which growth restarts, often when trees are still flooded (Schöngart et al. 2002). Growth rates are highest in the beginning of the terrestrial phase just after the flooding recedes and stops once the trees get flooded due to anoxic conditions around the roots (Schöngart et al. 2005). The terrestrial phase starts at the peak of the dry season in the wet floodplain site and during wet-dry season transition at the moist floodplain site. This species forms annual rings that follow the annual cycle of the flood-pulse of the rivers (Schöngart et al. 2005, Assahira et al. 2017).

### Tree ring sampling and isotopes analysis

For each *C. odorata* tree we cut a disc, and 10 mm cores were extracted from *M. acaciifolium* trees. One of the selected *C. odorata* trees is part of a published oxygen isotopes chronology that has been validated by radioncarbon dating (Baker et al. 2017). Tree rings were microscopically identified by wood anatomical features. For two samples of each species (one sample per site; see Figure 2), 9–11 rings were cut into thin segments of 0.02–3 mm for the intra-annual high resolution analysis. Very thin segments of 0.02 mm were cut using a core microtome (Gärtner and Nievergelt 2010). On average, rings were separated into 10–22 sections parallel to ring boundaries, although for a few very narrow rings only five sections could be cut. For *M. acaciifolium*, six additional samples were cut into three even-sized segments for the intra-annual medium resolution analysis. This was done to assess the representativeness of the floodplain trees for the general patterns in these environments. Three of these six additional samples are from the moist floodplain site and the other three are from a wet floodplain site located 500 km upstream of the wet floodplain site shown in Figure 2.

Cellulose was extracted from the wood using the Brendell et al. (2000) method, except for *C. odorata* from moist terra firme site where cellulose was extracted following Wieloch et al. (2011). Only the carbon isotope series from the west Amazonian floodplain was based on wholewood. Samples were freeze-dried and weighed in a precision balance to pack 0.5 ± 0.05 mg of samples in silver capsules for δ^18^O analysis and 1 ± 0.1 mg in tin capsules for δ^13^C analysis. Isotope analysis was done at the University of Leicester using an Isotopes Mass Spectrometer (Sercon 20-20 IRMS, Sercon IRMS, Crewe-UK) with precision of 0.15‰.

### Climate data

Local monthly precipitation, vapour pressure, temperature and cloud cover data for all sites were obtained from Climate Research Unit (CRU TS 4.00 0.5° resolution). Daily river stage data from the sites in Colombia and Bolivia were obtained from the nearest river gauging stations through the Hidroweb portal (http://www.snirh.gov.br/hidroweb/) from the Brazilian National System of Hydric Resources Information (SNIRH). As there were no local station data available for the site in Peru, we used instead monthly river data from the virtual river gauging station data from The Environmental Research Observatory (ORE) Geodynamical, Hydrological and Biogeochemical control of erosion/alteration and material transport in the Amazon basin (HYBAM).

The δ^18^O-data for precipitation were obtained from the Global Network of Isotopes in Precipitation and in the Global Network of Isotopes in River (GNIP and GNIR), accessed through the Water Isotopes System for Data Analysis, Visualization and Electronic Review (WISER, http://nds121.iaea.org/wiser/index.php). For the Bolivia site, we complemented this with monthly precipitation δ^18^O data (M. Gloor and R.J. Brienen, unpublished data).

Seasonal changes in climate were calculated for the same calendar years of the analysed tree rings at each site. Figure 2 shows the seasonal changes in monthly precipitation, inundation (floodplain sites only), VPD, cloud cover and rainfall δ^18^O for all studied sites.

### Data analysis

For each site, seasonal changes in intra-ring δ^18^O_TR_ and δ^13^C_TR_ were predicted using the available climatic data (see Climate data) from the site-specific growing seasons. Site-specific growing seasons were defined for each tree using the available information on growth rhythms for the *M. acaciifolium* (Schöngart et al. 2002) and *C. odorata* (Dünisch et al. 2003, Costa et al. 2013). These vary considerably between the terra firme (wet site: September–June; moist site: October–April) and floodplain sites (wet site: May–November; moist site: April–October). The model inputs used for the tree-ring isotopes predictions are presented in Table S2 available as Supplementary Data at *Tree Physiology* Online.

Covariation between observed isotope records was assessed using Pearson correlation coefficient. To assess the effect of inter-annual variation in climate on tree ring isotopes, we calculated mean isotope values for the complete ring. We then related the inter-annual variation in δ^13^C and δ^18^O for the full ring to local temperature, rainfall and cloud cover during the entire growing season of the trees. We did not consider VPD for these analyses, as the available data may not be accurate enough at the inter-annual level. In addition to local climate variables, we also considered Amazon basin-wide precipitation, which has been shown to influence local precipitation δ^18^O (Baker et al. 2016). Amazon basin-wide precipitation was calculated as the spatially integrated mean precipitation for the hydrological basin (see Baker et al. 2016). These analyses were done for the four trees with high intra-annual resolution.

In order to further explore the effects of seasonal climate variation on tree ring isotopic composition, we also calculated the dry season length and climate means over moving periods of 2–8 months across the trees’ growing seasons. Dry season length was defined as in Marengo et al. (2001) and calculated using daily rainfall data from Tropical Rainfall Measuring Mission (TRMM 3B42 0.25° resolution). These climate means were correlated with mean isotope values for the whole ring and with the mean isotope values for three intra-ring segments. For these analyses, the six *M. acaciifolium* oxygen series with medium intra-ring resolution were also included.

All analyses were done using the data analysis tool R, version 3.2.3.

## Results

Predicted δ^13^C_TR_ and δ^18^O_TR_ patterns for each site showed different contributions from seasonal changes in δ^18^O_sw_, VPD and temperature, and from estimated responses of *g*_sW_ and *g*_m_. For δ^18^O_TR_ most of the predicted variation comes from δ^18^O_sw_, but leaf water enrichment caused by changes in VPD and *g*_sW_ responses also contribute significantly to predicted δ^18^O_TR_ for the two trees growing at the drier sites (i.e., the moist floodplain and moist terra firme site; see Figure 2f, l, r and x and Figure S2 available as Supplementary Data at *Tree Physiology* Online). Using two different assumptions for effective pathlength (L) we also note that pathlength significantly affects the Péclet effect, especially under low relative humidity (see Figure S3 available as Supplementary Data at *Tree Physiology* Online, see also Kahmen et al. 2008, Holloway-Phillips et al. 2016). For our predictions however, we did not vary pathlength, as we had no species-specific data on pathlengths, and predicted contributions of leaf water enrichment to δ^18^O_TR_ thus reflects only site differences in VPD and *g*_sW_ (not pathlength difference).

For δ^13^C_TR_, *g*_sC_ responses to VPD contributed to most of the predicted δ^13^C_TR_ variations in all trees, except for the *C. odorata* from the wet terra firme site, which showed weak δ^13^C_TR_ variations (Figure 2g, m, s and y). Temperature effects over *g*_m_ also contributed significantly to the predicted δ^13^C_TR_ patterns for *M. acaciifolium* the moist floodplain site—where seasonal temperature variations were biggest—but showed little contribution for the other trees (see Figure S4 available as Supplementary Data at *Tree Physiology* Online).

**Figure 4.**
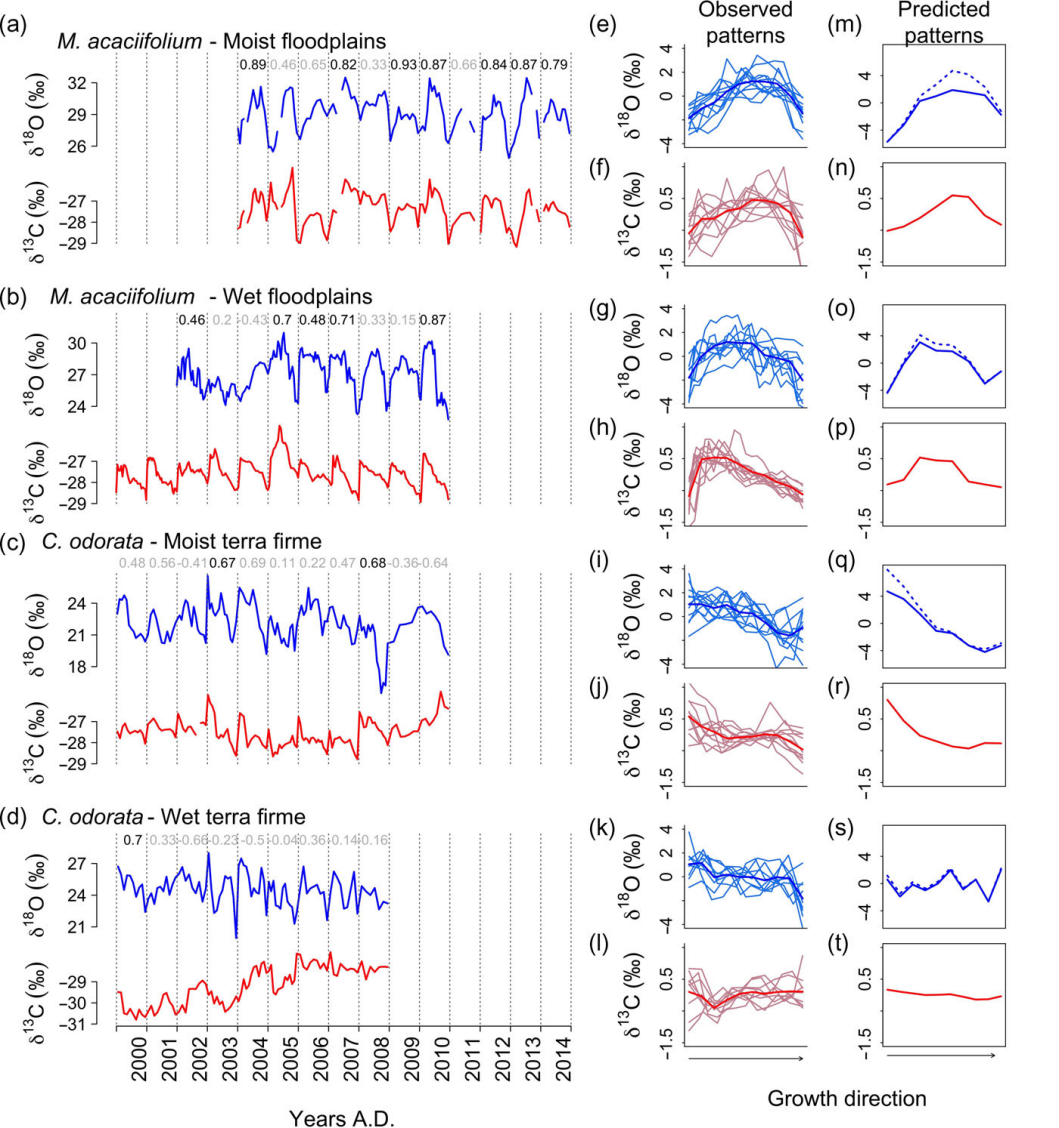
Observed high resolution series of δ^18^O_TR_ (blue lines) and δ^13^C_TR_ (red lines) (left panels), and observed (middle panels) and predicted (right panels) intra-annual cycles for the four study trees. The values at the top of the left side panels indicate the correlation coefficients between the δ^18^O and δ^13^C variations within each ring, with significant values in black. The solid blue and red lines in middle panels are the mean intra-annual cycles over all rings. Solid blue line in right panels represent the effects of only source water δ^18^O and stippled blue line indicates the added effects of leaf water enrichment. Note that the scale of the tree-ring δ^18^O predictions is larger than that for the observations (see the Discussion section for details). δ^18^O and δ^13^C values are from α-cellulose, except for the δ^13^C series from the wet floodplain, which is from wholewood.

Observed within-ring δ^18^O_TR_ variation matches the predicted patterns very well in all four trees with high intra-ring resolution (Figure 4—blue lines), although the within-ring amplitude was about two times lower for the observed patterns (4–5‰) compared with predictions (~10‰; see Table 1). The observed δ^13^C_TR_ patterns matches predictions quite well in the two *C. odorata* trees from the terra firme sites and for the *M. acaciifolium* tree from the moist floodplain site (Figure 4b and c, and right panels—red lines), but less well for the *M. acaciifolium* tree from the wet floodplain sites (Figure 4a and d, and right panels—red lines). The observed average amplitude for δ^13^C_TR_ is similar to predictions (~1‰). δ^13^C_TR_ in the initial section of individual rings was frequently higher than predicted in the *M. acaciifolium* from the wet floodplain and in some years for the *C. odorata* from moist terra firme, both of which show pronounced δ^13^C_TR_ increases of up to 2‰ across ring boundaries (Figure 4b and c—red lines).

**Table 1.** Summary the general isotopes sampling and isotopic patterns within rings for each tree. FP, floodplain; TF, terra firme.

	*Macrolobium acaciifolium*		*Cedrela odorata*	
	Moist FP	Wet FP	Moist TF	Wet TF
δ^18^O_TR_				
Total number of samples	129	196	128	90
Number of rings	11	9	11	9
Avg. samples per ring	12	22	12	10
Mean observed variation within rings variation (max – min, ‰)	4.33 ± 1.63	5.24 ± 1.46	4.61 ± 1.42	4.33 ± 1.75
Mean predicted variation within rings (max – min, ‰)	10.6	8.45	11.9	4.94
Avg. correlation between within-ring patterns	0.52	0.31	0.32	0.22
δ^13^C_TR_				
Total number of samples	129	228	129	90
Number of rings	11	10	11	9
Avg. samples per ring	12	23	12	10
Mean observed variation within rings (max – min, ‰)	1.86 ± 0.9	1.84 ± 0.56	1.41 ± 0.61	1.27 ± 0.28
Mean predicted variation within rings (max – min, ‰)	1.2	0.85	1.65	0.35
Avg. correlation between within-ring patterns	0.33	0.64	0.23	0.07
δ^18^O–δ^13^C correlations				
Avg. corr. Coef.	0.73	0.38	0.22	0.03
Number of correlated patterns within different rings	7	5	2	1

Comparison of δ^13^C_TR_ and δ^18^O_TR_ time series showed strong common features for some trees but less so for others. Within-ring δ^13^C and δ^18^O cycles in the floodplain trees were correlated in several years, especially at the moist site (Figure 4a and Table 1). In contrast, the terra firme trees only showed significant correlations between δ^18^O_TR_ and δ^13^C_TR_ in those rings with exceptionally large within-ring variations (Figure 4c and Table 1).

We also note that the δ^18^O time series of the two terra firme trees showed very similar patterns both on short and longer time scales (see Figure S5 available as Supplementary Data at *Tree Physiology* Online).

**Figure 5.**
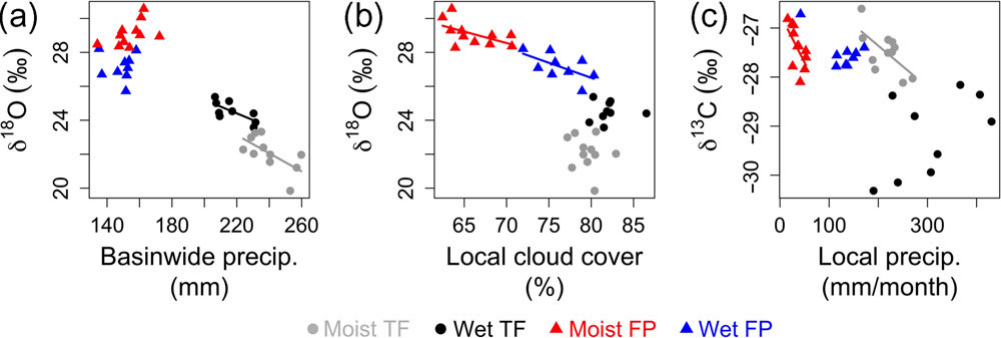
Summary of the climate effects on inter-annual variation in δ^18^O_TR_ and δ^13^C_TR_. Relation between δ^18^O_TR_ and basin-wide precipitation (a), δ^18^O_TR_ and local cloud cover (b) and δ^13^C_TR_ and local precipitation (c). Mean climate variables were calculated over trees’ respective growing seasons (see Materials and methods). Regression lines show significant (*P* < 0.05) relationships between isotopes and climate. δ^18^O and δ^13^C values are from α-cellulose, except for the δ^13^C series from the wet floodplain, which is from wholewood.

The regression analysis between climate variables and the tree-ring isotopes series revealed various significant correlations. For the two moist sites (terra firme and floodplain), we found that mean inter-annual ring δ^13^C was negatively correlated with local precipitation during the driest period of their growing seasons (Figure 5). No climatic effects on tree ring δ^13^C were found for the trees from the wet sites. Inter-annual mean ring δ^18^O variation of the two *M. acaciifolium* trees from the floodplain sites was positively correlated with cloud cover during the growing season (*P* < 0.1), while δ^18^O of the *C. odorata* trees from the terra firme sites was negatively correlated with Amazon-wide precipitation amounts (Figure 5).

Additional significant correlations with climate variables were found for specific ring segments and for shorter periods of 4–8 months within the growing season, showing the same overall pattern described above. Here we show correlations for sequentially moving windows of 4 and 6 months duration, as these summarize the patterns observed for both longer and shorter time spans (see Figure S6 available as Supplementary Data at *Tree Physiology* Online). These analyses included three additional *M. acaciifolium* trees per site (see Figure S7 available as Supplementary Data at *Tree Physiology* Online). For the *M. acaciifolium* trees from the wet flooplain site, average tree ring δ^18^O was also higher when the dry season started earlier (corr. Coef = 0.59, *P* < 0.05) or was longer (corr. Coef = 0.64, *P* < 0.05). Finally, for all trees the isotopic composition of specific ring segments showed generally correlations with more climate variables compared with mean ring isotopic time-series.

## Discussion

### High-resolution intra-ring variation in δ^13^C and δ^18^O

Our results show that the observed intra-annual δ^13^C_TR_ patterns agree reasonably well with the predicted δ^13^C_TR_ patterns, and that δ^13^C_TR_ variations thus follow seasonal variation in VPD (since our model does not include NSC reserve remobilization). For the floodplain species *M. acaciifolium*, within-ring variation in δ^13^C_TR_ shows strong positive peaks in δ^13^C_TR_ in the initial or middle sections of the ring. These δ^13^C_TR_ peaks coincide with the drier conditions at the initial and middle periods of the growing season at the wet and moist site, respectively. In the moist terra firme tree, we find decreases in δ^13^C_TR_ from the initial to the final sections of the ring, which is consistent with steadily decreasing water stress as the rainy season progresses. As predicted, we find a rather constant mean δ^13^C_TR_ at the wet terra firme site due to generally humid conditions throughout the growing season of this tree (Figure 4). Thus, observed δ^13^C_TR_ patterns are largely consistent with expected plant stomatal responses to changes in VPD during the growing season of each tree. Our model also included temperature effect on *g*_m_. This effect slightly improved the match between predicted and observed δ^13^C_TR_ patterns for the *M. acaciifolium* trees from the moist floodplain site (Figure 4 and see Figure S4 available as Supplementary Data at *Tree Physiology* Online). For this tree, we also noted that seasonal temperature variations predict relatively large variation in δ^13^C_TR_ due to mesophyll conductance without requiring any change in *g*_sC_ (see Figure S4e available as Supplementary Data at *Tree Physiology* Online). While this effect is only predicted in one tree, it shows that the temperature effects on *g*_m_ could significantly influence seasonal variations in δ^13^C_TR_ and may be more important to C-isotope discrimination than generally assumed (Griffiths and Helliker 2013, von Caemmerer and Evans 2015).

In addition to climate effects, we expected to observe effects of NSC remobilization on intra-annual δ^13^C_TR_. Both investigated species completely change their leaves annually and remain leafless for several weeks (*C. odorata*) or some days (*M. acaciifolium*) (Schöngart et al. 2002, Dünisch et al. 2003). We thus expected sharp increases in δ^13^C_TR_ values at tree ring boundaries in the records of all trees, related to starch-dependent stem growth before initial leaf flush. We only observe clear sharp increases in δ^13^C_TR_ for *M. acaciifolium* from the wet floodplain site (Figure 4b). As these sharp increases occurred exactly across the ring boundaries, and as peak δ^13^C_TR_ due to climate was predicted to occur later in the season (see Figure 4p), we suspect these patterns may be due to use of NSC at the start of the growing season. We also find smaller peaks early in the rings of *C. odorata* from the moist site. *Cedrela odorata* is indeed known to use starch from the previous year for stem growth at the beginning of the growing season (Dünisch and Puls 2003). Thus, the data provide some evidence for carbon remobilization effects, but these effects are not consistent between the two trees of each species and seemingly unrelated to deciduousness. Other studies in the tropics similarly show that the effect is not always evident in deciduous species (Poussart et al. 2004, Ohashi et al. 2009, Fichtler et al. 2010) and can also be seen in evergreen trees (Schleser et al. 2015). More work is needed to understand carbon remobilization effects on tree ring δ^13^C_TR_.

Observed intra-annual δ^18^O_TR_ patterns match very well our predictions for all four trees. They follow the changes in precipitation δ^18^O and predictions for leaf water enrichment over the respective growing season for each tree (see Figure S2 available as Supplementary Data at *Tree Physiology* Online). The observed amplitude, however, is much lower (4–5‰) than that predicted by the two combined effects (10–15‰). It is also lower than the single effect of precipitation δ^18^O, but matches the amplitude arising from leaf water enrichment (see Figure S2 available as Supplementary Data at *Tree Physiology* Online). For the source water contribution one would indeed expect a somewhat lower amplitude in δ^18^O_TR_ compared with rainfall δ^18^O, as plants use soil water, which is expected to show less seasonal variation. This is because water from successive precipitation events mix in a larger reservoir in the soil (Brooks et al. 2010, Evaristo et al. 2015), and this effect is not included in our predictions. δ^18^O_TR_ results alone thus cannot discern between leaf level processes and source water influences. Further analysis of the climate signals and covariation between δ^18^O_TR_ and δ^13^C_TR_ in the next sections provide more insights about the dominant drivers of δ^18^O_TR_ for each of the studied trees.

### Relations between inter-annual variation in tree ring isotopes and climate

δ^13^C_TR_ in three trees reflected local precipitation during the driest part of the growing season (see Figure S6 available as Supplementary Data at *Tree Physiology* Online). This is consistent with known effects of water availability on leaf ^13^C-isotope discrimination as trees close their stomata to prevent water loss when soil moisture and relative humidity are low (Farquhar and Sharkey 1982). It is also consistent with observations of negative correlations between δ^13^C and precipitation for tropical trees growing at relatively dry sites (e.g., Gebrekirstos et al. 2009, Fichtler et al. 2010, Brienen et al. 2012). The only tree for which we do not find a relationship with precipitation in the expected direction is the *C. odorata* tree growing at the wet terra firme site. This is probably because the site is so wet (precipitation rarely drops below 100 mm per month). We have no explanation for the observed positive correlation between precipitation and δ^13^C in this tree.

The relations between climate variables and tree-ring oxygen isotopes suggests that different dominant drivers control inter-annual variation in δ^18^O in the two species; in the *M. acaciifolium* floodplain trees δ^18^O covaries with temperature, cloud cover, local precipitation and dry season length, while for the two *C. odorata* terra firme trees δ^18^O covaries with basin-wide precipitation amount (Figure 5 and see Figure S6 available as Supplementary Data at *Tree Physiology* Online). Temperature, cloud cover and precipitation may reach increasingly stressful levels when the dry season is longer, and probably affect δ^18^O in floodplain trees primarily via their effects on evaporative leaf water enrichment above source water δ^18^O. Leaf water enrichment depends on water vapour pressure of the atmosphere, air temperature, isotopic composition of atmospheric water, leaf temperature and stomatal conductance (Barbour et al. 2000, Barbour and Barbour 2007, Kahmen et al. 2008; see Figure 1). Surprisingly, tree-ring δ^18^O for both floodplain trees correlates most strongly with cloud cover, and this pattern is still consistent in the analysis including the six trees with medium intra-ring resolution oxygen series (see Figure S6 available as Supplementary Data at *Tree Physiology* Online). This may be because reduced cloud cover during the dry season may lead to higher leaf temperatures (Doughty and Goulden 2009), lower air humidity (Quaas 2012) and thus raised leaf-to-air vapour pressure difference (VPD), and consequently reductions in stomatal conductance (Lloyd and Farquhar 2008). Overall, these results are consistent with theory and experimental studies of the effects of local moisture conditions over leaf water enrichment and δ^18^O_TR_ variations (Barbour and Farquhar 2000, Barbour et al. 2000, Barbour 2007, Cernusak et al. 2016).

For the terra firme trees, which grow during the wet season, tree ring δ^18^O is mainly influenced by Amazon basin-wide precipitation (Figure 5), and less by local climate (see Figure S6 available as Supplementary Data at *Tree Physiology* Online). This suggests that the tree ring δ^18^O_TR_ signal is a precipitation δ^18^O_TR_ signal. Precipitation δ^18^O_TR_ in turn is the result of the cumulative effects of all precipitation events upwind from the sites. This is because heavy water isotopes are gradually removed at each precipitation event during moisture transport from the tropical Atlantic to the study sites, so precipitation will be more depleted in δ^18^O during years with more rain over the Amazon, assuming incoming δ^18^O_TR_ does not vary from year to year (Salati and Vose 1984, Vimeux et al. 2005, Villacís et al. 2008). These results are consistent with the known precipitation δ^18^O influence on δ^18^O_TR_ in *C. odorata* tree rings (Brienen et al. 2012, Baker et al. 2015, 2016). In line with this is the coherence in δ^18^O_TR_ patterns within rings (intra-annually) observed for the two terra firme sites, *C. odorata trees* from Bolivia and Peru, which are ~1000 km apart (see Figure S5 available as Supplementary Data at *Tree Physiology* Online). These results demonstrate that source water is the dominant influence on tree ring δ^18^O for these *C. odorata* trees. We note, however, that δ^18^O for the *C. odorata* tree at the moist site showed some (weak) correlations with local precipitation during the start of the growing season (see Figure S6 available as Supplementary Data at *Tree Physiology* Online), consistent with weak local effects observed in longer tree-ring δ^18^O_TR_ series from the same site in Bolivia (Brienen et al. 2012). Also consistent with this is predicted contribution of leaf enrichment to δ^18^O_TR_ in initial ring sections of this tree, caused by relatively dry conditions during the start of the growing season (Figure 4q). In all, for these *C. odorata* trees, source water is the dominant signal, with possible weak influences of local precipitation at the start of the growing season for the moist trees. Although we do not have replications for these trees, these results are consistent with previous studies which show that mean inter-annual tree-ring δ^18^O variations of *C. odorata* trees from different sites (including our same moist terra firme site) are driven by source water δ^18^O (Brienen et al. 2012, Baker et al. 2015, 2016).

### Dual-isotope analysis

A striking property of the isotope records is the strong intra- and inter-annual covariation between carbon and oxygen isotopes in the floodplain *M. acaciifolium* trees (see Figure 4, Table 1 and see Figure S8 available as Supplementary Data at *Tree Physiology* Online). This covariation is particularly strong for the *M. acaciifolium* tree from the moist floodplain site with highly similar features in the intra-annual patterns (Figure 4a), and also good correlations between mean annual δ^13^C_TR_ and δ^18^O_TR_ (see Figure S8 available as Supplementary Data at *Tree Physiology* Online). Such strongly covarying patterns suggest a common driver. The one common process for δ^13^C_TR_ and δ^18^O_TR_ is the response of stomatal conductance to water status of the soil–plant continuum and VPD. These results thus strongly support the climate-δ^18^O_TR_ analysis for this species, which suggests that variation in δ^18^O_TR_ is primarily controlled by leaf ^18^O enrichment, and that the initial source water signal is dampened in the final tree ring δ^18^O signal.

In contrast, in the two terra firme trees, the δ^18^O_TR_ and δ^13^C_TR_ records of each tree are generally uncorrelated (Figure 4c and d), indicating that they are not both primarily influenced by stomatal conductance effects on leaf ^13^C-discrimination and/or on leaf ^18^O enrichment. This decoupling of variation in δ^13^C_TR_ and δ^18^O_TR_ in this species could be due to a lack of control of stomatal conductance on δ^13^C_TR_, δ^18^O_TR_ or both. As we observe a weak negative relation between δ^13^C_TR_ and precipitation only at the moist site and an opposite relation at the wet site, control of stomatal conductance on leaf ^13^C-discrimination seems to be weaker in *C. odorata*. While this is one possible explanation, a more plausible reason for the lack of covariation between δ^18^O_TR_ and δ^13^C_TR_ is that δ^18^O_TR_ in this species mainly records variation in source water δ^18^O and only weak local climate effects, as we showed here (see Figure S6a available as Supplementary Data at *Tree Physiology* Online) and also in Brienen et al. (2012). This lack of ^18^O leaf enrichment signals in δ^18^O_TR_ for *C. odorata* may be due to either low levels of leaf enrichment above the source water in the leaf, or because any occurring leaf isotope enrichment is not transferred to the final tree ring δ^18^O in this species because of extensive exchange of leaf exported sugar with stem water during cellulose synthesis (Sternberg 2008). The former explanation, a lack of leaf enrichment, could be due to species-specific leaf traits, such as higher leaf transpiration rates and/or longer effective pathlengths reducing strongly the effect of back diffusion on average leaf δ^18^O (Kahmen et al. 2008, Cernusak and Kahmen 2013; see also Figure S3 available as Supplementary Data at *Tree Physiology* Online).

Apart from purely species-specific effects, differences in the leaf enrichment contributions to δ^18^O_TR_ for the four trees could also be influenced by variation in trees’ growing season humidity. Predictions from isotope theory, confirmed by lab experiments (Roden and Ehleringer 1999, Roden and Farquhar 2012), are that leaf water enrichment above the plant source water is small for trees growing in humid conditions, and increases with increasing VPD (Barbour et al. 2002, 2004, Roden and Siegwolf 2012). Interestingly, for each species, the tree growing at the drier sites (moist floodplain and moist terra firme) showed stronger correlations between intra-annual δ^18^O_TR_ and δ^13^C_TR_ variations than the tree growing at the wetter site (wet floodplain and wet terra firme). This provides some indication that growing season water availability/relative humidity may control the strength of the covariation between both stable isotopes in tree rings of tropical trees. More research is needed to assess how environmental conditions, specifically relative humidity, affect the strength of source water vs leaf enrichment signals in tree rings.

## Conclusions

We investigated δ^13^C and δ^18^O in cellulose of two Amazon terra firma and two floodplain trees located along a precipitation gradient. We show here that intra-annual variation in isotopes (δ^13^C_TR_ and δ^18^O_TR_) in four Amazon trees growing in different environments follow predictions based on isotope theory. Observed intra-annual variation in δ^13^C_TR_ agreed well with Farquhar’s model of leaf level ^13^C discrimination considering stomatal responses to seasonal variation in VPD and temperature effects on mesophyll conductance, and suggest a direct transfer of climate signals from leaf to tree ring. We do also find some signatures of post-photosynthetic carbon remobilization effects on δ^13^C_TR_, which are especially clear in the wet floodplain tree and to a lesser degree in the moist terra firme tree. Intra-annual variation in δ^18^O_TR_ closely matched seasonal variation in source water and the predicted effects of leaf water enrichment due to variation in VPD.

The inter-annual variation in δ^13^C_TR_ was controlled by local precipitation for trees at the drier growing conditions, but not at the wettest site. Inter-annual variation in δ^18^O_TR_ showed different controls in the two species; the floodplain species *M. acaciifolium* recorded variation in leaf water enrichment in response to local climate (cloud cover), while the terra firme species *C. odorata* recorded source water δ^18^O variation, which is controlled by large-scale rainout signals (i.e., basin-wide precipitation).

The four trees showed differences in the degree of covariation between δ^13^C_TR_ and δ^18^O_TR_ with the strongest covariation in the floodplain tree experiencing the driest growing conditions and lowest covariation for the wet terra firme tree. Higher covariation in the drier sites are most likely the result of stomatal responses to humidity affecting both isotopes in the same way. The four trees represent a continuum from entirely source water dominated δ^18^O_TR_ signal (in the wettest site) to primarily leaf level process dominated δ^18^O_TR_ signal (in the driest site). Our data cannot reveal whether variation in control of δ^18^O_TR_ signals is caused by species-specific differences in physiology (*C. odorata* vs *M. acciifolium*), or truly reflects a dominant influence of VPD gradients.

Our results provide some clear insights, but also raise new questions. Firstly, we showed that across the four trees, δ^13^C_TR_ reflected primarily photosynthetic carbon-discrimination responses to humidity and temperature. We also found signatures of carbon remobilization effects, but surprisingly, these were not linked to species phenology. More research under what circumstances carbon remobilization occurs and how it affects δ^13^C_TR_ will help interpreting tree ring isotope signals. Secondly, our results suggest that δ^18^O_TR_ can be controlled by very different processes, source water δ^18^O variation vs leaf water enrichment, but it remains unclear which process dominates when and under what circumstances. Difference in the controls on δ^18^O_TR_ have profound implications for the interpretation of δ^18^O_TR_ in palaeo-climatic and plant physiological studies. For example, source water δ^18^O signals may record large-scale rainout information over continents (Brienen et al. 2012, Baker et al. 2016), or hurricane influences on coastal sites (Miller et al. 2006), while leaf water enrichment signals are expected to reflect climate variation of a much more local nature via VPD (i.e., Kahmen et al. 2008).

## Supplementary Material

Supplementary Data
