# Supplementary material for: Contrasting controls on tree ring isotope variation for Amazon floodplain and terra firme trees
Source: Tree Physiol. 2019 Apr 6;39(5):845–60. doi: 10.1093/treephys/tpz009 (PMC6594573; doi:10.1093/treephys/tpz009)
Supplement: Supplementary Data [file tpz009_si_20181204.docx]

## *Tree Physiology* – Supporting Information

Article title: **Contrasting controls on tree ring isotope variation for Amazon floodplain and terra firme trees**

Authors: Bruno Barçante Ladvocat Cintra, Manuel Gloor, Arnoud Boom, Jochen Schongart Giuliano Maselli Locosselli and Roel Brienen

The following Supporting Information is available for this article (10 pages):
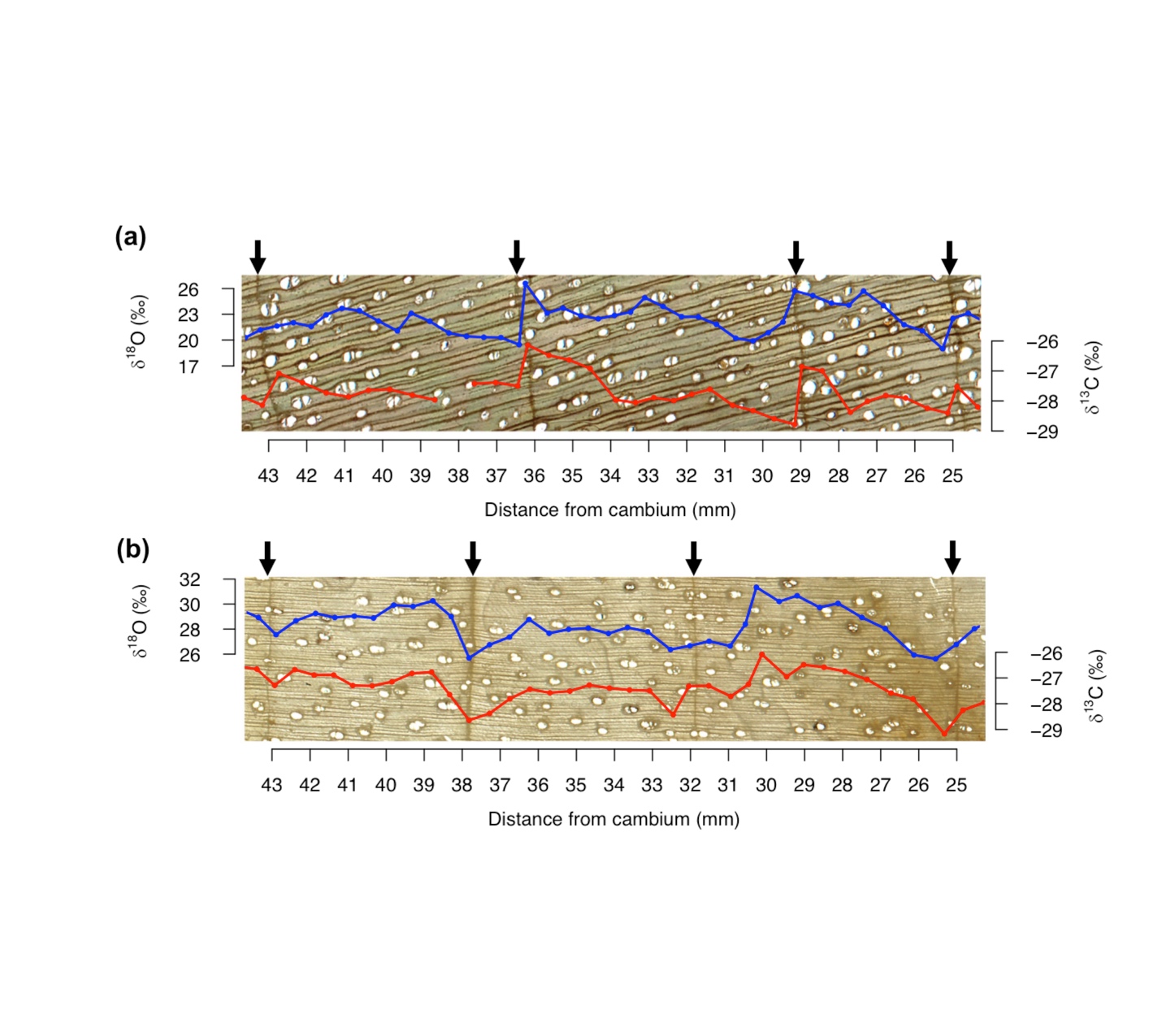


**Fig. S1** **Anatomical wood structure of Macrolobium acaciifolium (panel a) and Cedrela odorata (panel b) with in blue the intra-ring resolution δ^18^O_TR_ and in red the δ^13^C_TR_ (red lines) series.** Arrows indicate the ring boundaries. δ^18^O and δ^18^O values shown are from α-cellulose.


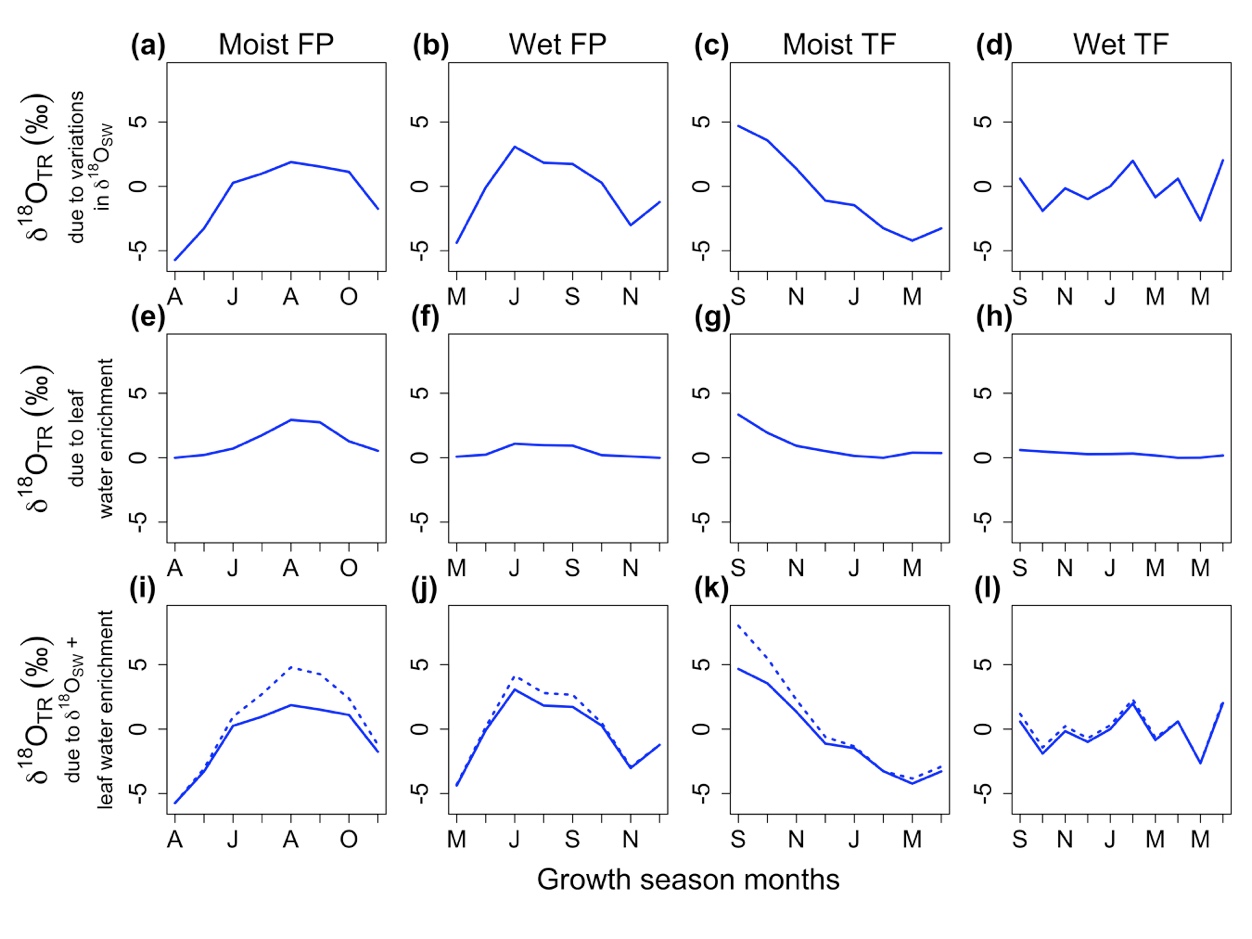


**Fig. S2 Predicted seasonal patterns of tree ring δ^18^O (δ^18^O_TR_) due to only source water δ^18^O effect (top panels), only leaf water enrichment (middle panels), and the combined effect (lower panels).** The stippled line in the lower panels indicates the leaf water enrichment above source water δ^18^O (solid line). Panels (a,e,i) give the predictions for the Moist Floodplain site, (b,f,j) for the Wet Floodplain site, (c,g,k) for the Moist Terra firme site and (d,h,l) for the Wet terra firme site.

**
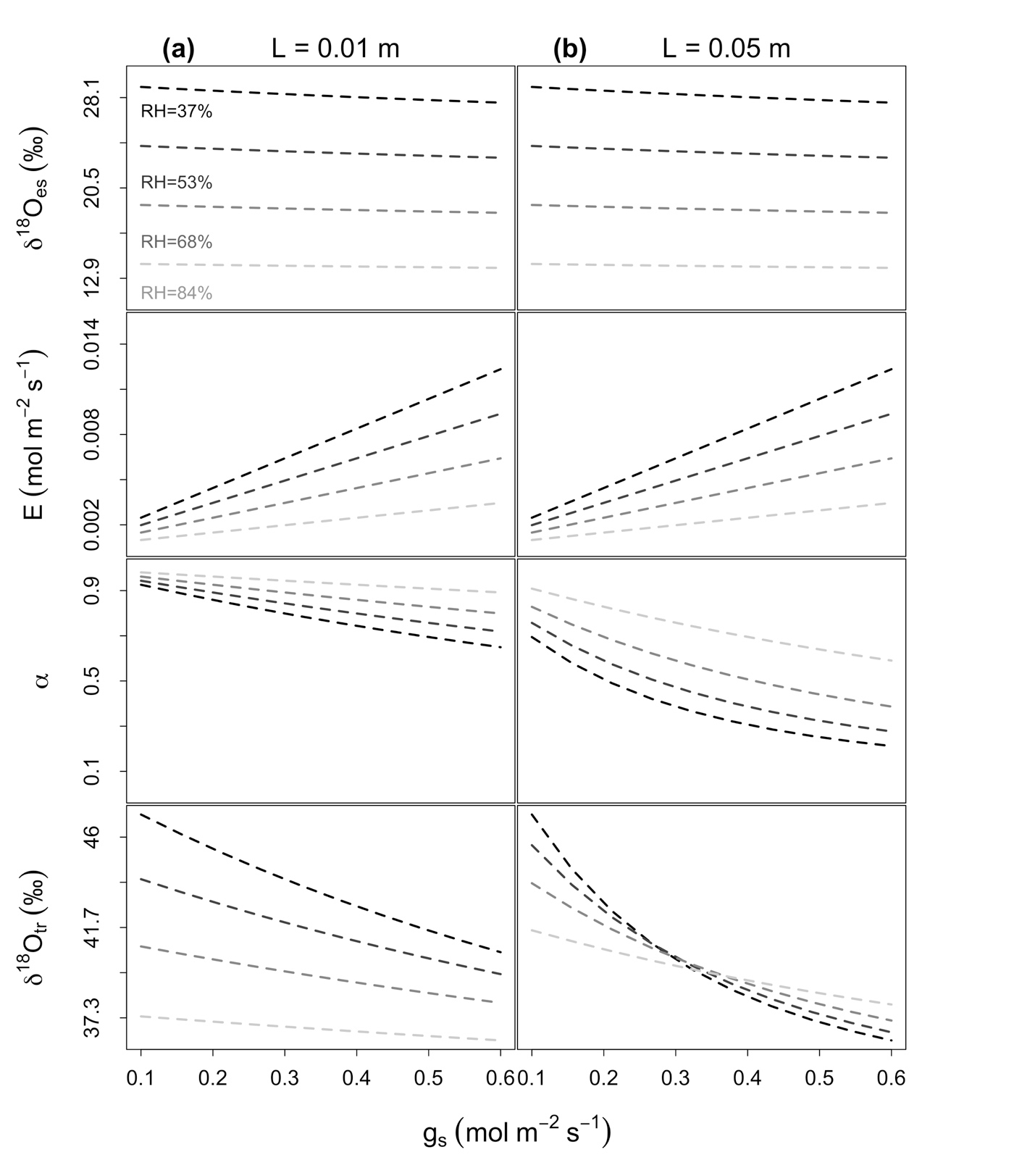
**

**Fig. S3** **Predicted effects of stomatal conductance (g_s_) on leaf δ^18^O at four different levels of relative humidity (RH) and for two different path lengths.** Left panels show effects for short path lengths of L=0.01, and right panels for L=0.05m, and from top to bottom showing the modelled relationship between g_s_ and d18O of water at the sites of evaporation (δ^18^O_es_) (panel a,b), g_s_ and leaf transpiration (E) (panel c,d), g_s_ and “admixture” of source water with water from the sites of evaporation (alpha=$\left( \frac{\left( 1-e^{-℘} \right)}{℘} \right)$ (panel e,f), and g_s_ and mean leaf water δ^18^O (δ^18^O_lw_) (panel g,h).


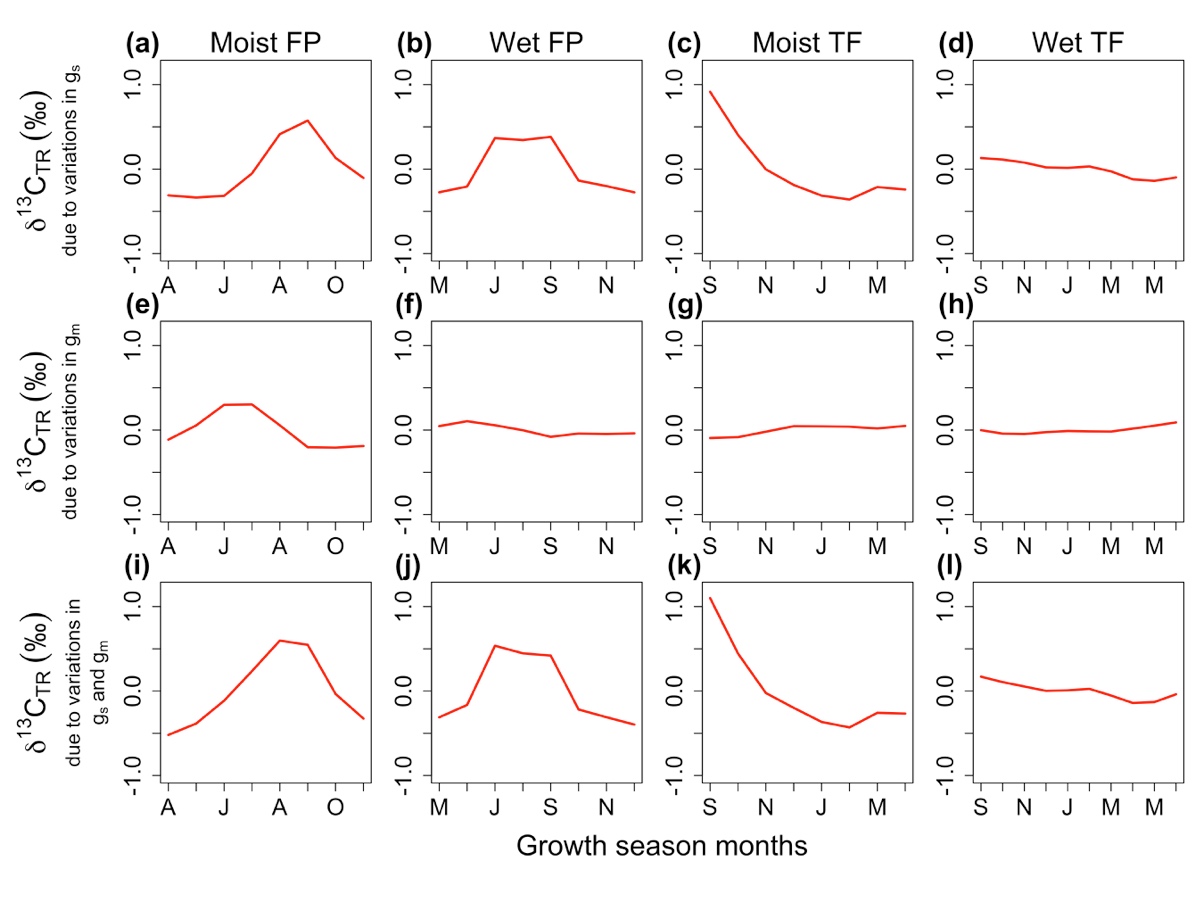


**Fig. S4 Predicted seasonal patterns of tree ring δ^13^C (δ^13^C_TR_) due to only *g_s_* variations (top panels), only *g_m_* variations (middle panels), and the combined effect (lower panels).** Panels (a,e,i) give the predictions for the Moist Floodplain site, (b,f,j) for the Wet Floodplain site, (c,g,k) for the Moist Terra firme site and (d,h,l) for the Wet terra firme site.


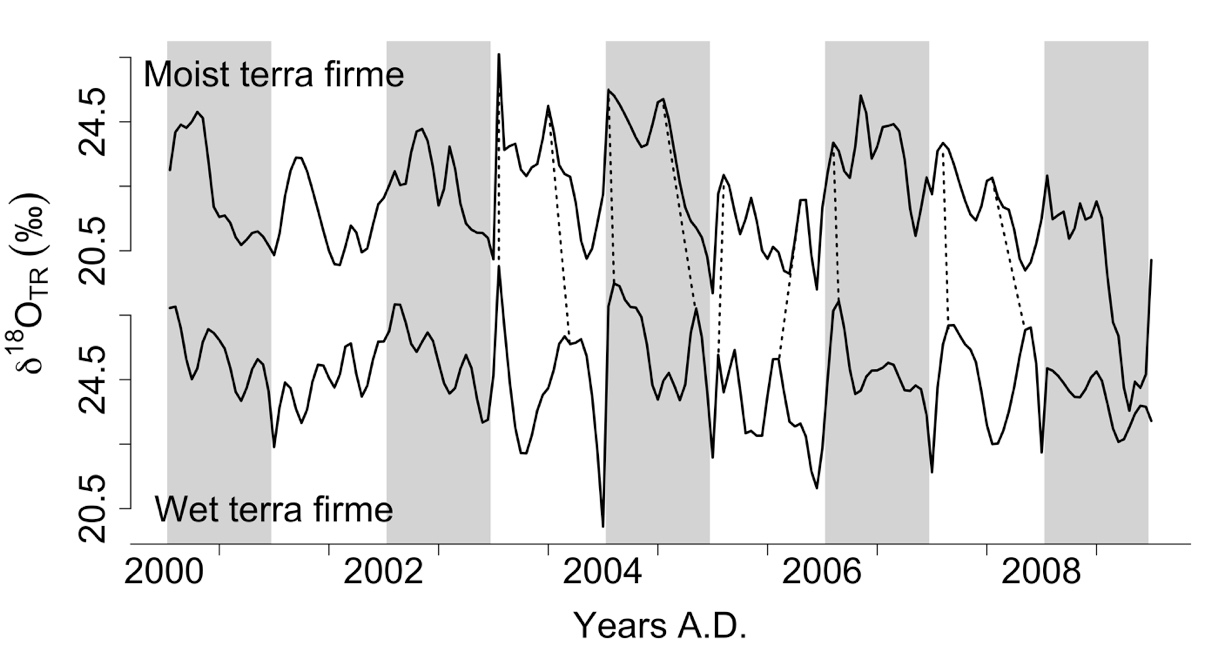


**Fig. S5 Covariation of tree ring δ^18^O patterns in Cedrela odorata trees from the two firme sites.** Dashed lines indicate common feature in the two series. The sites are 1000 km apart, located in northern Bolivia and Peru (see map in Fig, 1).


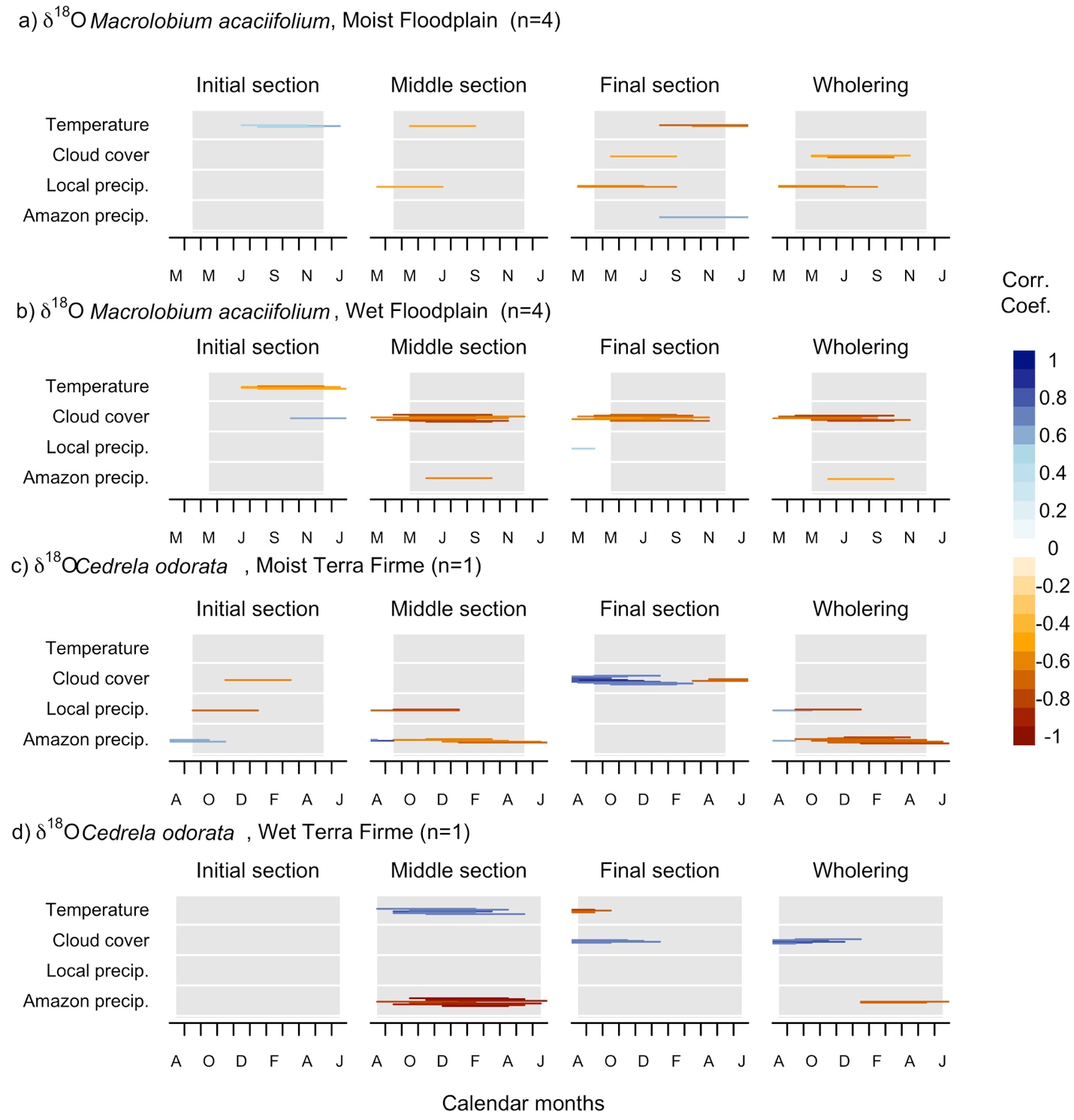


**Fig. S6 Heatmap of the correlations of the inter-annual isotope series with climate variables for the four sites.** Panels a-d show the correlations observed for δ^18^O_TR_, panels e-h show the correlations for δ^13^C_TR_. Correlations are shown for isotopes values of three ring sections and for the whole ring. Pearson correlations were performed using annual mean isotope values against climate variables averaged over running periods of 4 and 6 months lengths. Only statistically significant correlations (p < 0.05) are shown. Grey shaded areas indicate the current growing season (right) and the previous growing season (left).


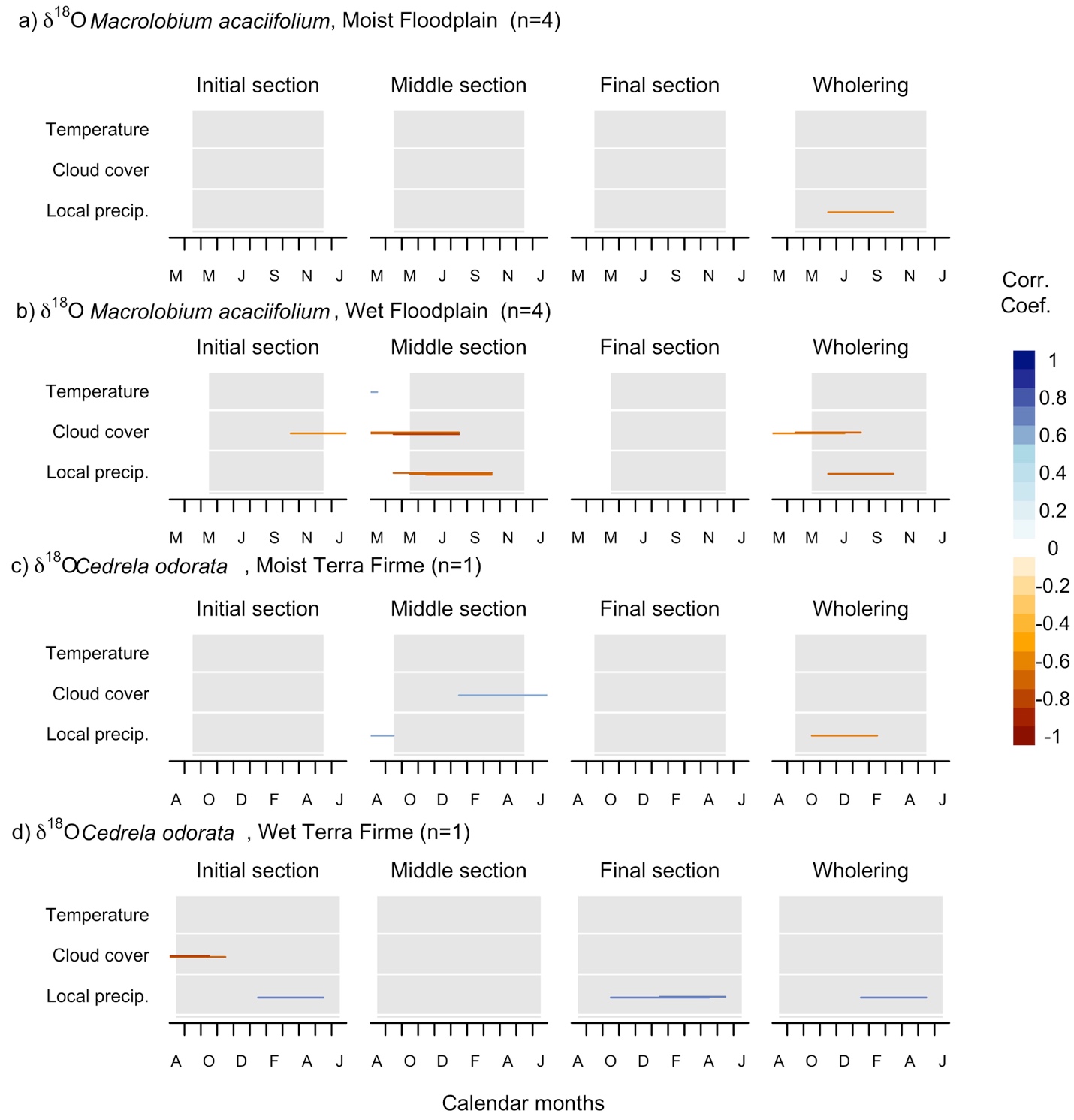


**Fig. S6 Continued.**

**
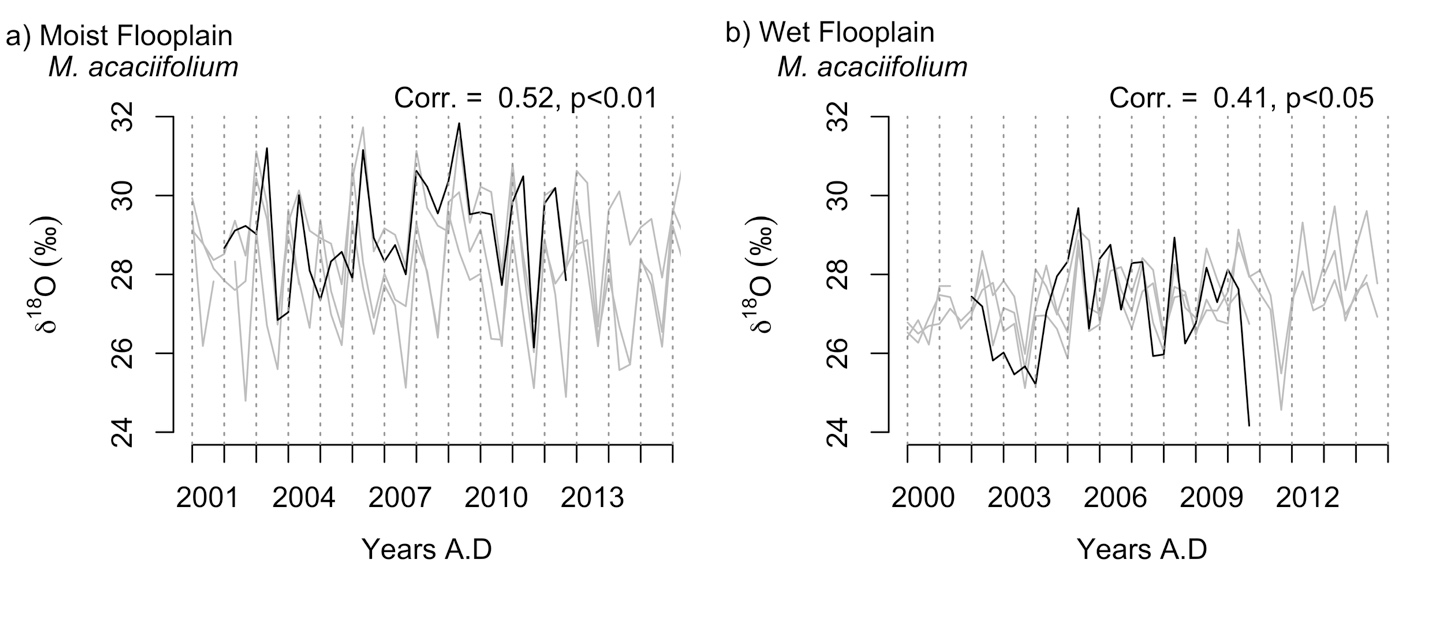
**

**Fig. S7 Time series of intra-annual tree-ring oxygen isotopes series (δ^18^O_TR_) of Macrolobium acaciifolium with 3 samples per ring.** a) 4 trees from the Moist floodplain site including the tree shown in the main text (black) (eps=0.78, mean interseries correlation = 0.47). b) 4 trees including the tree from the Wet Floodplain site shown in the main text (black) and 3 trees from a wet floodplain site approximately 500km upstream (eps= 0.77, mean interseries correlation=0.43). Mean interseries correlations are the mean of all pairwise correlations between all series. The coefficients shown in the top of each panel indicate the correlations between the trees with intra-ring high resolution data shown in the main text and the average series of the other 3 trees with 3 samples per ring. >> This figure is now shown in the Supplementary information of the manuscript and the six additional series shown here were included in the analyses shown in SI Figure S6.


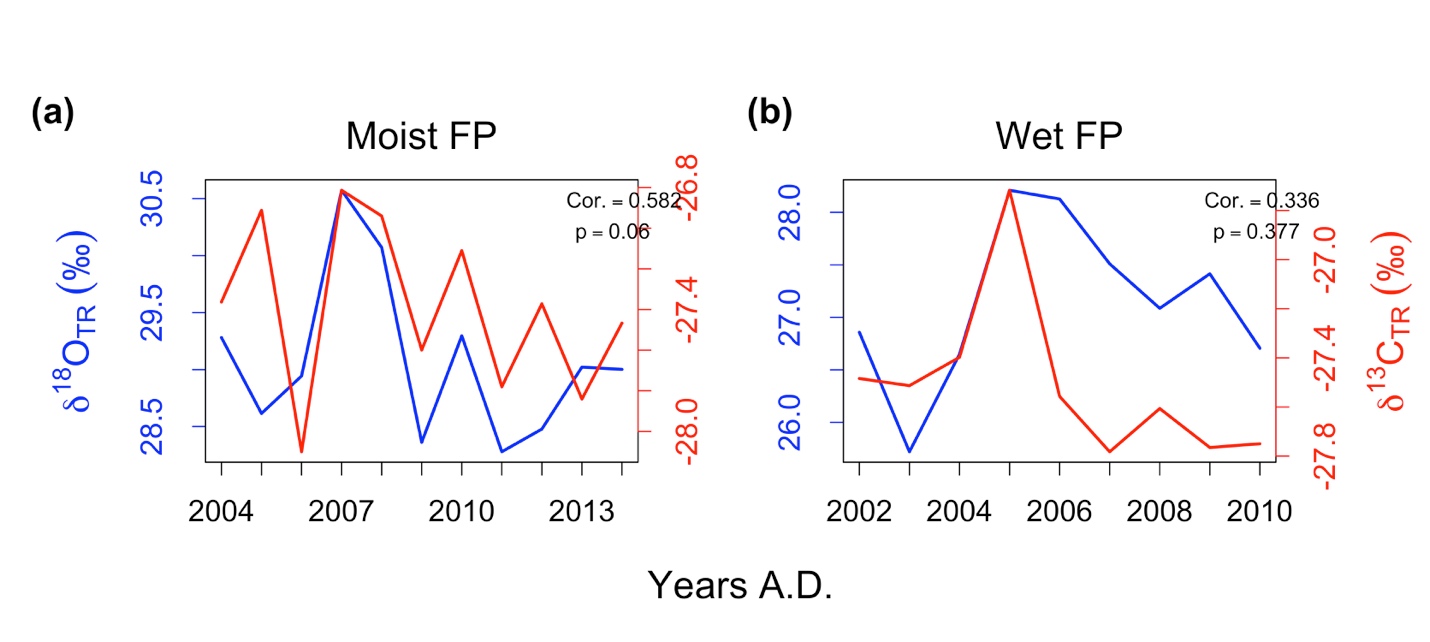


**Fig. S8 Time series of annual δ^18^O and δ^13^C for the two floodplain trees.** Numbers indicate the Pearsons correlation coefficient and p-level. δ^18^O and δ^13^C values are from α-cellulose, except for the δ^13^C series from Wet FP, which is from wholewood.

**Table S.1: List of symbols and abbreviations used in this study.**

| Symbol | Description | Units |
| --- | --- | --- |
| $\delta^{13}C_{plant}$ | Carbon isotope composition in plant tissues | $‰$ |
| ${}^{13}C_{atm}$ | Carbon isotopes composition of atmospheric CO_2_ | $‰$ |
| $\delta^{18}O_{es}$ | Oxygen isotope com position at the evaporative sites in the leaf (e.g. stomata and mesophyll spaces) | $‰$ |
| $\delta^{18}O_{lw}$ | Oxygen isotope composition of the leaf lamina water | $‰$ |
| $\delta^{18}O_{\mathrm{tr}}$ | Average oxygen isotope composition of tree ring cellulose | $‰$ |
| $\delta^{18}O_{sw}$ | Oxygen isotope composition of rainfall water | $‰$ |
| $\delta^{18}O_{air}$ | Oxygen isotope composition atmospheric vapour | $‰$ |
| ${}^{+}$ | Equilibrium fractionation during evaporation of H_2_^18^O | $‰$ |
| ${}_{k}$ | Kinetic fractionation factor during diffusion of H_2_O from stomata to the atmosphere | $‰$ |
| ${}_{wc}$ | Fractionation of oxygen during synthesis of sugars in the leaf | $‰$ |
| $a$ | Fractionation during CO_2_ diffusion through stomata | $‰$ |
| $b$ | Fractionation due to isotopic discrimination during carboxylation | $‰$ |
| $c_{i}$ | Leaf internal CO_2_ partial pressure | $\mathrm{kPa}$ |
| $c_{a}$ | Atmospheric CO_2_ partial pressure | $\mathrm{kPa}$ |
| $e_{a}$ | Atmospheric vapour pressure | $\mathrm{kPa}$ |
| $e_{i}$ | Leaf internal saturated vapour pressure | $\mathrm{kPa}$ |
| $e_{sat}$ | Saturated vapour pressure | $\mathrm{kPa}$ |
| $VPD$ | Atmospheric vapour pressure deficit | $\mathrm{kPa}$ |
| ${VPD}_{0}$ | Average vapour pressure deficit over the growing season | $\mathrm{kPa}$ |
| $P$ | Atmospheric pressure | $\mathrm{kPa}$ |
| $p_{x}p_{ex}$ | Proportion of enriched water in the cell (*p_x_*) and proportion of oxygen exchanged with water in the cell during cellulose synthesis (*p_ex_*) | $\%$ |
| $℘$ | Péclet number | $\mathrm{Dimentionless}$ |
| $W$ | Leaf lamina diameter | $m$ |
| $L$ | Path length from leaf veins to stomata through the mesophyll | $m$ |
| $D$ | Diffusion rate of H_2_^18^O in water | $m^{2}s^{-1}$ |
| $u$ | Velocity of the advective transport of water from leaf veins to stomata | $m s^{-1}$ |
| $U$ | Wind speed | $m s^{-1}$ |
| $C$ | Molar concentration of water | $\mathrm{mol}m^{-3}$ |
| $E$ | Leaf transpiration | $\mathrm{mol}m^{-2}s^{-1}$ |
| $A$ | Assimilation rate of CO_2_ during photosynthesis | $\mathrm{mol}m^{-2}s^{-1}$ |
| $g_{s}$ | Leaf stomatal conductance | $\mathrm{mol}m^{-2}s^{-1}$ |
| $g_{s0}$ | Maximum stomatal conductance | $\mathrm{mol}m^{-2}s^{-1}$ |
| $g_{b}$ | Leaf boundary layer conductance | $\mathrm{mol}m^{-2}s^{-1}$ |
| $T_{leaf}$ | Leaf temperature = air temperature | $\mathrm{Celsius}$ |
| $T_{air}$ | Air temperature | $\mathrm{Celsius}$ |

**Table S2: Mechanistic models and inputs used in the calculation of the expected patterns of isotopic variation within tree-rings.**

| Carbon Isotopes fractionation |
| --- |
| $\boldsymbol{\delta}^{\boldsymbol{13}}\boldsymbol{C}_{\boldsymbol{plant}}\boldsymbol{\approx}\boldsymbol{\delta}^{\boldsymbol{13}}\boldsymbol{C}_{\boldsymbol{atm}}\boldsymbol{-a}\left( \frac{\boldsymbol{c}_{\boldsymbol{a}}\boldsymbol{-}\boldsymbol{c}_{\boldsymbol{i}}}{\boldsymbol{c}_{\boldsymbol{a}}} \right)\boldsymbol{-b}\left( \boldsymbol{1-}\frac{\boldsymbol{c}_{\boldsymbol{a}}\boldsymbol{-}\boldsymbol{c}_{\boldsymbol{i}}}{\boldsymbol{c}_{\boldsymbol{a}}} \right)\boldsymbol{\sim-27\pm3}‰$ (Farquhar *et al.*, 1989) |
| $a=4.4 ‰$; $b=27 ‰$ (Farquhar *et al.*, 1989); ${}^{13}C_{atm}=7.7 ‰$ (NOAA) |
| $\boldsymbol{c}_{\boldsymbol{i}}\boldsymbol{=}\boldsymbol{c}_{\boldsymbol{a}}\boldsymbol{-}\left( \frac{\boldsymbol{A}}{\boldsymbol{g}_{\boldsymbol{s}}} \right)\boldsymbol{P}\mathrm{kPa}$ (Farquhar *et al.* 1989) |
| $P=101.325\mathrm{kPa}$; $c_{a}=0.04154325\mathrm{kPa}$ (NOAA, ~410ppm in July 2018) ; $A={1x10}^{-6} \mathrm{mol}m^{-2}s^{-1}$ (assumed) |
| $\boldsymbol{g}_{\boldsymbol{s}}\boldsymbol{=}\boldsymbol{g}_{\boldsymbol{s}\boldsymbol{0}}\left( \frac{\boldsymbol{1}}{\boldsymbol{1}\boldsymbol{+}\frac{\boldsymbol{VPD}}{\boldsymbol{VPD}_{\boldsymbol{0}}}} \right)\boldsymbol{\sim}\boldsymbol{0}\boldsymbol{.}\boldsymbol{2}\boldsymbol{\pm}\boldsymbol{1} \mathbf{mol}\mathbf{m}^{\mathbf{-2}}\mathbf{s}^{\mathbf{-1}}$ (assumed) |
| $g_{s0} =0.3 \mathrm{mol}m^{-2}s^{-1}$ (assumed); $VPD=e_{sat}- e_{a}\sim0.8\pm0.4 \mathrm{kPa}$ (CRU TS 4.00); $e_{a}\sim2.6\pm0.4 \mathrm{kPa}$ (CRU TS 4.00); $e_{sat}= 0.61121 exp\left( 18.678-\frac{T_{leaf}}{234.5} \right)\left( \frac{T_{leaf}}{257.14+T_{leaf}} \right)\sim3.4\pm0.3 \mathrm{kPa}$; $T_{leaf}=T_{air} \sim26\pm2 \mathrm{Celsius}$ (CRU TS 4.00); ${VPD}_{0}=growing season long term mean \sim1\pm0.5$ kPa (CRU TS 4.00) |
| Oxygen Isotopes Fractionation |
| $\boldsymbol{\delta}^{\mathbf{18}}\mathbf{O}_{\mathbf{tr}}\boldsymbol{=}\boldsymbol{\delta}^{\mathbf{18}}\mathbf{O}_{\mathbf{sw}}\boldsymbol{+}\left( \boldsymbol{\delta}^{\mathbf{18}}\mathbf{O}_{\mathbf{lw}}\boldsymbol{-}\boldsymbol{\delta}^{\mathbf{18}}\mathbf{O}_{\mathbf{sw}} \right)\left( \boldsymbol{1-}\boldsymbol{p}_{\boldsymbol{x}}\boldsymbol{p}_{\boldsymbol{ex}} \right)\boldsymbol{+}{}_{\boldsymbol{wc}} ‰$ (Barbour & Farquhar, 2000) |
| $\delta^{18}O_{sw}=-5\pm6 ‰$ GNIP, Brienen & Gloor (unpublished data); $\delta^{18}O_{air}=\delta^{18}O_{sw}‰$ (assumed); ${}_{wc}=27 ‰$ (Cernusak & Kahmen, 2013) |
| $\boldsymbol{\delta}^{\mathbf{18}}\mathbf{O}_{\boldsymbol{lw}}\boldsymbol{=}\boldsymbol{\delta}^{\mathbf{18}}\mathbf{O}_{\mathbf{sw}}\boldsymbol{+}\left( \boldsymbol{\delta}^{\mathbf{18}}\mathbf{O}_{\mathbf{es}}\boldsymbol{-}\boldsymbol{\delta}^{\mathbf{18}}\mathbf{O}_{\mathbf{sw}} \right)\boldsymbol{*}\frac{\left( \boldsymbol{1-}\boldsymbol{e}^{\mathbf{-℘}} \right)}{\mathbf{℘}} ‰$ |
| $℘\equiv\frac{Advection}{Diffusion} =\frac{u}{D/L}$; $L=0.02 m$ (assumed); $D=2.3 x {10}^{-9} m^{-2}s^{-1}$; $u=\frac{E}{C} m^{2}s^{-1}; E=g_{s} \frac{VPD}{atm}\sim0.003 \mathrm{mol}m^{-2}s^{-1}$; $C=55 x {10}^{3} \mathrm{mol}m^{-3}$ |
| $\boldsymbol{g}_{\boldsymbol{s}}\boldsymbol{=}\boldsymbol{g}_{\boldsymbol{s}\boldsymbol{0}}\left( \frac{\boldsymbol{1}}{\boldsymbol{1}\boldsymbol{+}\frac{\boldsymbol{VPD}}{\boldsymbol{VPD}_{\boldsymbol{0}}}} \right)\boldsymbol{\sim}\boldsymbol{0}\boldsymbol{.}\boldsymbol{2}\boldsymbol{\pm}\boldsymbol{1} \mathbf{mol}\mathbf{m}^{\mathbf{-2}}\mathbf{s}^{\mathbf{-1}}$ (assumed) |
| $g_{s0} =0.3 \mathrm{mol}m^{-2}s^{-1}$ (assumed); $VPD=e_{sat}- e_{a}\sim0.8\pm0.4 \mathrm{kPa}$ (CRU TS 4.00); $e_{a}\sim2.6\pm0.4 \mathrm{kPa}$; $e_{sat}= 0.61121 exp\left( 18.678-\frac{T_{leaf}}{234.5} \right)\left( \frac{T_{leaf}}{257.14+T_{leaf}} \right)\sim3.4\pm0.3 \mathrm{kPa}$; $T_{leaf}=T_{air} \sim26\pm2 \mathrm{Celsius}$; ${VPD}_{0}=growing season long term mean \sim1\pm0.5$ kPa (CRU TS 4.00) |
| $\boldsymbol{\delta}^{\boldsymbol{18}}\mathbf{O}_{\boldsymbol{es}}\mathbf{=}\left( \boldsymbol{\delta}^{\boldsymbol{18}}\mathbf{O}_{\boldsymbol{sw}}\boldsymbol{+}\boldsymbol{\varepsilon}_{\boldsymbol{k}} \right)\left( \frac{\boldsymbol{e}_{\boldsymbol{i}}\boldsymbol{-}\boldsymbol{e}_{\boldsymbol{a}}}{\boldsymbol{e}_{\boldsymbol{i}}} \right)\boldsymbol{+}\boldsymbol{\varepsilon}^{\boldsymbol{+}}\boldsymbol{+}\boldsymbol{\delta}^{\boldsymbol{18}}\mathbf{O}_{\boldsymbol{a}}\left( \frac{\boldsymbol{e}_{\boldsymbol{a}}}{\boldsymbol{e}_{\boldsymbol{i}}} \right) ‰$ (Dongmann *et al.*, 1974) |
| ${}^{+}=2.644-3.206\left( \frac{{10}^{3}}{T_{K}} \right)+1.534\left( \frac{{10}^{6}}{{T_{K}}^{2}} \right)\sim9.57 ‰$ (Bottinga & Craig, 1969); $T_{K}=T_{leaf}=299\pm4 \mathrm{Kelvin}$; ${}_{k}=\frac{32g_{s}^{-1}+21g_{b}^{-1}}{g_{s}^{-1}+g_{b}^{-1}} \sim26.5\pm5 ‰$ (Farquhar *et al.*, 1989); $g_{b}=0.0105\left( \frac{U}{W} \right)^{-0.5}=1.2\pm0.5 \mathrm{mol}m^{-2}s^{-1}$ (McDermitt, 1990; Motzer *et al.*, 2005); $U=0.5 m s^{-1}$(assumed); $W=0.05$ (*Cedrela odorata*); $W=0.01$(*Macrolobium acaciifolium*) |
